# Insect taxonomy can be difficult: a noctuid moth (Agaristinae: *Aletopus imperialis*) and a geometrid moth (Sterrhinae: *Cartaletis dargei*) combined into a cryptic species complex in eastern Africa (Lepidoptera)

**DOI:** 10.7717/peerj.11613

**Published:** 2021-06-25

**Authors:** Pasi Sihvonen, Leidys Murillo-Ramos, Niklas Wahlberg, Axel Hausmann, Alberto Zilli, Michael Ochse, Hermann S. Staude

**Affiliations:** 1 Finnish Museum of Natural History “Luomus”, University of Helsinki, Helsinki, Finland; 2 Departamento de Biologia, Universidad de Sucre, Sincelejo, Sucre, Colombia; 3 Department of Biology, Lund University, Lund, Sweden; 4 SNSB Zoologische Staatssammlung München, Munich, Germany; 5 Department of Life Sciences, Natural History Museum, London, United Kingdom; 6 Waldstraße 51, Weisenheim am Berg, Germany; 7 Caterpillar Rearing Group (CRG), LepSoc Africa, Magaliesburg, South Africa

**Keywords:** Agaristinae, Biodiversity, Cryptic species, Geometridae, Molecular, Morphology, Noctuidae, Sterrhinae, Systematics, Tanzania

## Abstract

The systematic position of a large and strikingly coloured reddish-black moth, *Cartaletis dargei* Herbulot, 2003 (Geometridae: Sterrhinae) from Tanzania, has remained questionable since its description. Here we present molecular and morphological evidence showing that *Cartaletis dargei* only superficially resembles true *Cartaletis* Warren, 1894 (the relative name currently considered a junior synonym of *Aletis* Hübner, 1820), which are unpalatable diurnal moths superficially resembling butterflies, and that it is misplaced in the family Geometridae. We transfer it to Noctuidae: Agaristinae, and combine it with the genus *Aletopus* Jordan, 1926, from Tanzania, as *Aletopus dargei* (Herbulot, 2003) **(new combination)**. We revise the genus *Aletopus* to contain three species, but find that it is a cryptic species complex that needs to be revised with more extensive taxon sampling. Our results demonstrate the difficulties in interpreting and classifying biological diversity. We discuss the problems in species delimitation and the potential drivers of evolution in eastern Africa that led to phenotypic similarity in unrelated lepidopteran lineages.

## Introduction

The phenomenon that unrelated insects resemble each other superficially is widespread. Occasionally, the similarity happens to a degree that organisms can be separated only by detailed investigation of morphology or DNA. The phenomenon has fascinated workers ever since Bates (1862) first noticed this and many speculative articles have been published, but the actual drivers of the phenomenon are often poorly studied. Those may include amongst others convergent evolution, mimicry, crypsis and aposematism (Scoble, 1995; Meyer, 2006; Chiocchio et al., 2020).

Mimicry, an evolved resemblance between unrelated organisms, is a widespread phenomenon in Lepidoptera. A well-known example is the Batesian-Müllerian mimicry complex involving the North American Viceroy butterfly (*Limenitis archippus*), which is somewhat different in different parts of its distribution, closely matching the colouration patterns and display behaviour of locally coexisting species such as the Monarch butterfly (*Danaus plexippus*), the Queen butterfly (*Danaus gilippus*) and the Soldier butterfly (*Danaus eresimus*) (Ritland, 1995; Pohl et al., 2009). Many unrelated diurnal Lepidoptera in Central and South America are part of mimicry rings (a group of species within a local community having an aposematic signal in common), sharing for instance the striking black-and-yellow *Cyllopoda* pattern (Sihvonen et al., 2020), or glasswing pattern that is shown by Lepidoptera and Odonata (Corral-Lopez et al., 2020), or the complex “*Heliconius”* pattern (for instance (Meyer, 2006; Kronforst & Papa, 2015; Jiggins, 2017)).

Staude & Curle (1997) presented a functional view on the visual signals emanating from the wings of Afrotropical Lepidoptera. They noted that adult Lepidoptera, including nocturnal and diurnal species, follow four types of strategies as defense against predator, namely: visual (morphological), behavioural, acoustic and olfactory signals. Visual cues often aim to avoid predation by signaling chemical inedibility. Within the species that follow the visual strategy, “wing-tip signal” (species having light spots or a band towards the apex of the forewing on a dark background, causing a flashing at the end of each clap of the flying process) is perhaps the most widespread and is shown by very diverse unrelated lineages including diurnal Geometridae, Noctuidae: Agaristinae and Erebidae: Arctiinae.

One of the lineages possessing the wing-tip signal is *Aletis* Hübner, 1820 (=*Cartaletis* Warren, 1894) moths, which are diurnal geometrids that occur in sub-Saharan Africa. Their butterfly-like habitus may be the reason why these moths show a complex taxonomic history, and in biodiversity portals like iNaturalist they are called Monarch Loopers. The type species of genus *Aletis*, *A. helcita* (Linnaeus, 1763), described from tropical Africa, was originally combined with the butterfly genus *Papilio* (*Danaus*) (Linnaeus, 1763). These diurnal Lepidoptera were recognised to be geometrid moths by Prout (1929–35) and Janse (1933–35), who classified them in the subfamily Oenochrominae. Janse was the first to describe detailed morphological structures of *Aletis* (called at the time *Cartaletis*), including the male genitalia, tympanal organs, antennae and wing venation. Holloway (1996) noted that *Aletis* has genitalia structures typical of Sterrhinae: Scopulini, and therefore Aletini should be treated as a synonym of Scopulini. The most detailed account on *Aletis* morphology, so far, was provided by Sihvonen (2005), who carried out a phylogenetic analysis of Scopulini based on structural characters. In that work, both *Aletis* and *Cartaletis* were synonymised with *Scopula*, which is a genus of about 800 species mostly with nocturnal habits. Currently, based on molecular and morphological evidence, *Aletis* Hübner, 1820 is considered as a valid genus with *Cartaletis* Warren, 1894 as its junior synonym. This taxon is classified in Sterrhinae: Scopulini as sister to the *Problepsis* + *Isoplenodia* lineage (Sihvonen et al., 2020).

Both larvae and adult moths in *Aletis* (=*Cartaletis*) are brightly coloured and aposematic, and adults can attain wingspans of up to 70 mm, unlike their nocturnal relatives, which are cryptic in appearance and usually less than 30 mm in size (Sihvonen, 2005). The males are restricted to high-up in the tree canopy of tropical forests, whereas the females are mostly found in the understory where the host plant grows. The larvae have been reared on *Oxyanthus* (Rubiaceae) (Staude et al., 2020), containing toxic cyanogenic glycosides (Rockenbach, Nahrstedt & Wray, 1992).

*Cartaletis dargei* Herbulot, 2003 was described on the basis of two males from Tanzania: Rungwe Mission on 8th of March 2002, together with other Geometridae species (Herbulot, 2003). The publication is ‘authoritative’ in the sense that Herbulot did not justify the classification of *C. dargei* in Geometridae or in *Cartaletis*, neither did he provide diagnostic characters for the new species. Despite that, the publication fulfills the ICZN requirements of being available (ICZN, 1999, Article 13.1.1). Herbulot provided drawings of the male genitalia, but as said, he neither compared nor discussed the structures against other taxa.

**Figure 1:**
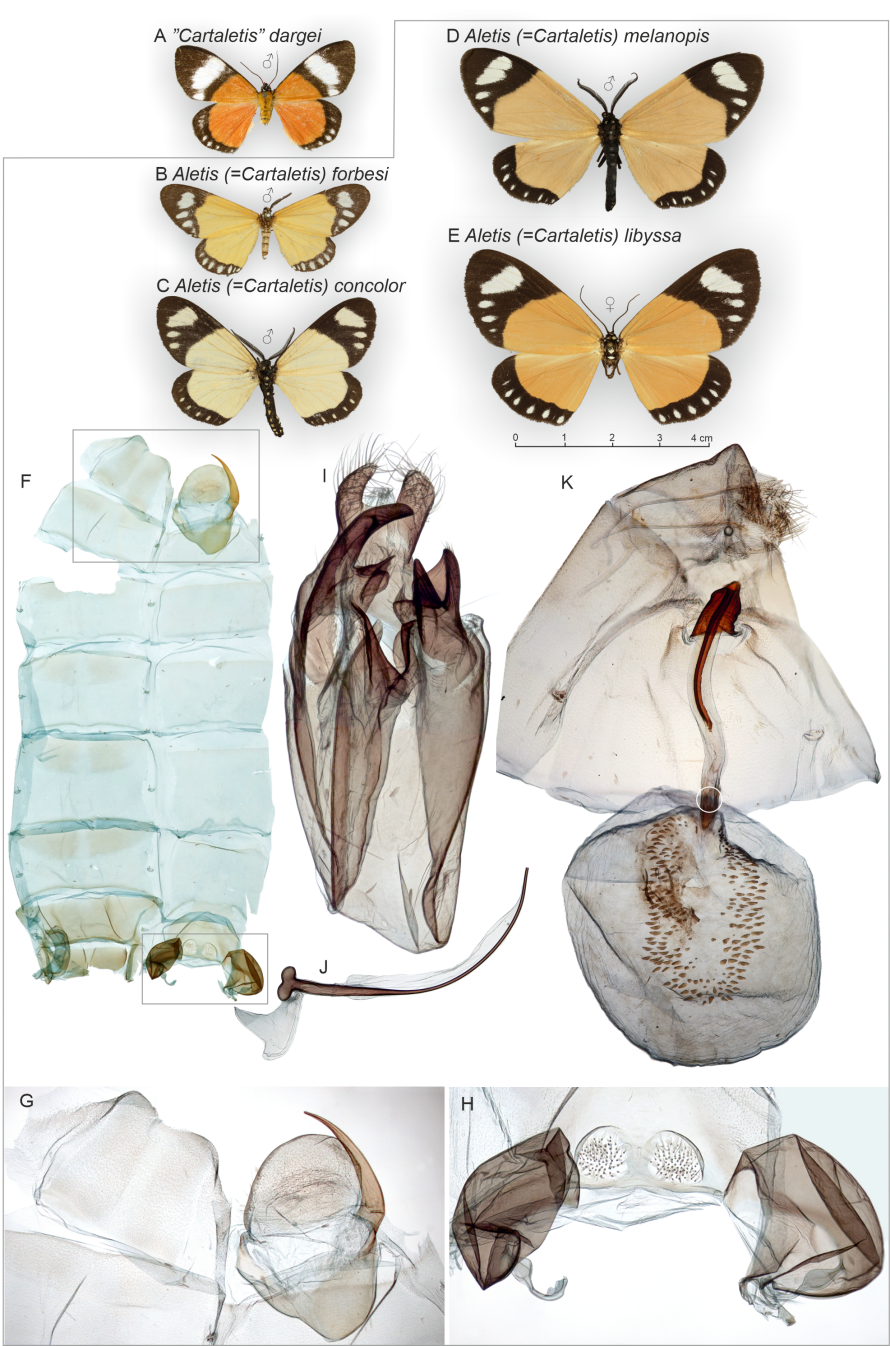
“*Cartaletis*” *dargei* (Noctuidae), external variation of selected *Aletis* (=*Cartaletis*) moths (Geometridae), and selected structures of *Cartaletis libyssa*. *Cartaletis libyssa* (E) is the type species of *Cartaletis* Warren, 1894, and its male abdomen (F) and genitalia structures (I–K) are illustrated. Male 8th segment (G) and tympanal organs with additional medial sclerotisations (H) are shown enlarged. White circle indicates the point of origin of the ductus seminalis in the female genitalia. (A) “*Cartaletis*” *dargei* Herbulot2003, male, holotype. Tanzania: Rungwe mission, Iisière forest, 1550 m, 8 Nov. 2002 (coll. ZSM, dissected 8042/Herbulot, barcoded ZSM Lep 58147. (B) *Aletis forbesi* (Druce, 1884), male, holotype. Nigeria: Banks of the Lower Niger (coll. NHMUK). (C) *Aletis concolor* Warren, 1905, male, syntype. South Africa: Zululand (coll. NHMUK). (D) *Aletis melanopis* Prout1929, male, holotype. Burundi: Kibira Forest, north end of Lake Tanganyika, 7000 ft (coll. NHMUK). (E) *Aletis libyssa* (Hopffer, 1858), female. Democratic Republic of Congo: Katanga district, March 1927 (dissected BMNH GEO 20468/Sihvonen, coll. NHMUK). (F) *Aletis libyssa*, male abdomen. South Africa, Barberton, near Josefdal, 28.XII.1991, 1700 m, mist forest, H.S.Staude leg., HSS db 4512 (Sihvonen_slide_2038, coll. ZSM). (G) *Aletis libyssa*, male 8th segment. Same data as F.(H) *Aletis libyssa*, male tympanal tympanal organs and additional medial sclerotisatons. Same data as F. (I) *Aletis libyssa*, male genitalia. Same data as F. (J) *Aletis libyssa*, male aedeagus. Same data as F. (K) *Aletis libyssa*, female genitalia. Lu[or w] at[or I or J]aba/ Katanga dist.,/ Congo Belge./ Mar. 1927.; Rothschild Bequest B.M. 1939-1. Democratic Republic of the Kongo: Katanga district, March 1927 (dissected BMNH GEO 20468/Sihvonen, coll. NHMUK).

Despite the superficial similarity between *Cartaletis dargei* and the original concept of *Cartaletis* (Fig. 1), a close examination reveals that they are unrelated. This is particularly obvious when the male genitalia of the *C. dargei* holotype, as illustrated in Herbulot (2003), are compared against corresponding structures of *Aletis* (=*Cartaletis*) (Fig. 1) and (Janse, 1933–35; Sihvonen, 2005). Herbulot dissected and mounted the abdomen of the holotype (deposited in the Zoologische Staatssammlung München ZSM), but it lacks segments A1–A3 which have remained attached at the pinned specimen. These abdominal segments are informative with regard to classification, for instance whether the specimen has tympanal organs on sternites A1–A2, which are diagnostic in the Geometridae (Minet & Scoble, 1999).

We present molecular and morphological data in a phylogenetic, comparative and diagnostic context to pinpoint the systematic position of *Cartaletis dargei* in Noctuidae: Agaristinae. We illustrate the relevant taxa in question, demonstrate that it is a cryptic species complex and a challenge from the taxonomic point of view, and provide diagnostic characters for the mentioned subfamily.

## Materials & Methods

### Taxon sampling and material repositories

Materials from the following collections were studied: ANIC—Australian National Insect Collection (CSIRO), Canberra, Australia; FMNH—Finnish Museum of Natural History, Helsinki, Finland; HSS—Research Collection of Hermann Staude, Magaliesburg, South Africa; NHMUK—Natural History Museum, London, United Kingdom; Ochse—Research Collection of Michael Ochse, Weisenheim am Berg, Germany; UOZM—University of Oulu Zoological Museum; ZSM—Zoologische Staatssammlung München (SNSB), Germany. In addition, literature and online sources on Lepidoptera were screened extensively. Table 1 summarizes the new molecular (genes) and morphological data (dissections) of the study, including the examined type material.

**Table 1:** Molecular and morphological data used in this study. Specimens are allocated to either *Aletopus dargei* group or *A. imperialis* group (see Results). In addition, molecular data from Zahiri et al. (2013) were used, see that publication for details. GenBank accession numbers and/or BIN numbers are also provided. New sequences produced in this study have sample IDs “Sihvonen DNA 184, 185, 188, 190″.

**Name and sex**	**Label data**	**Molecular data: sample ID, GenBank accession number/BIN number Morphological data: sample ID**	**coll.**
***”Cartaletis” dargei*** holotype m	Tanzanie: Rungwe mission, (1550 m) Iisièré forestière, 8-III-2002 Ph. Darge; Pr. No. 8042, C. Herbulot; Cartaletis, dargei, Hrblt, HOLOTYPE [red label]; BC ZSM Lep 58147; Photographed, for the project, “Geometridae, mundi”	**Molecular** BC ZSM Lep 58147: COI-begin - MW590952 **Morphology** Pr. No. ZSM G [1] 8042 C. Herbulot: Abdomen, genitalia	ZSM
***Aletopus dargei*** group m	Malawi: Chitipa district, Mughese forest, 6000 FT, 9°39′S 33°32′E, 9-16 Jan 2002, leg. R. J. Murphy	**Morphology** Sihvonen 2046: Abdomen, genitalia Sihvonen 2047: Wings SEM photos: Head	HSS
***Aletopus dargei*** group f	Tanzania: Milo. 1800 m, 10°00.30′S 34°38.09′E, 04-iii-2013, leg. ABRI MH-PW	**Molecular** Sihvonen DNA 184: COI-begin - MW549773 Wgl400 - MW548615 **Morphology** Sihvonen 2052: Abdomen, genitalia	HSS
***Aletopus dargei*** group m	Tanzania: Iringa region, Livingstone Mts., forét Sud de, Mlangali, 2070 m, 7-XI-2004, leg. Ph. Darge, 09°48.573′S 34°31.016′E	**Molecular** BC ZSM Lep 20051 COI-begin - HM376557-SUPPRESSED **Morphology** Sihvonen 2832: Abdomen, genitalia	ZSM
***Aletopus imperialis*** group m	Tanzania: West Usambara, Magamba Forest, 2015 m, 04°42.615′S 38°14.244′E, XII-2003, leg. Ph. Darge	**Molecular** Sihvonen DNA 185: COI-begin - MW549774 Wgl400 - MW548616 **Morphology** Sihvonen 2834: Abdomen, genitalia	ZSM
***Aletopus imperialis*** group m	Tanzania: West Usambara, Magamba Forest, 2000 m, 04°43.399′S 38°14.744′E, XII-2003, leg. Ph. Darge	**Molecular** Sihvonen DNA 188: COI-begin - MW549775 **Morphology** Sihvonen 2078: Abdomen, genitalia	ZSM
***Aletopus imperialis*** group f	Tanzania: Kombola, 2010	**Molecular** Sihvonen DNA 190: COI-begin - MW549776 Wgl400 - MW548617 **Morphology** Sihvonen 2833: Abdomen, genitalia	HSS
***Aletopus imperialis*** group m	Tanzania: West Usambara, Magamba Forest, 2000 m, 4°82′S 38°44′E, 01-Dec-2003, leg. Ph. Darge	**Molecular** BC ZSM Lep 42575 COI-begin - MW590953	ZSM
***Aletopus imperialis*** group m	Malawi: Chitipa district, Mughese forest reserve, 6000 FT, 09°39′S 33°32′E, 9-16-Jan-2002, R. J. Murphy	**Morphology** Sihvonen 2829: Abdomen, genitalia	Ochse
***Aletopus imperialis*** group m	Tanzania: Iringa, Ulembwe, 2070 m, 09°18.709′S, 34°38.078′E, 22-Dec-2008, leg. Ph. Darge	**Morphology** Sihvonen 2830: Abdomen, genitalia	Ochse
***Aletopus imperialis*** group m	Tanzania: Morogoro Region, Uluguru Mts., Bunduki Forest, Alt. 1275 m., 07°01.679′S, 37°37.945′E, 25-Jan-2008, Ph. Darge	**Morphology** Sihvonen 2831: Abdomen, genitalia	ZSM
***Aletopus imperialis*** f holotype	[Tanzania]: Usambara, Bungu, IX 1921; Type; Aletopus, imperialis, Type Jord., Nov. Zool. 1926; coll. Loveridge.; Noctuidae Brit. Mus. slide No. 8298; NHMUK 014198923	**Morphology** Noctuidae Brit. Mus. slide No. 8298: Abdomen and genitalia	NHMUK
***Aegocera tigrina*** m	Zambia: 40 km SE Mbala, 09°07′S 31°45′E, 1565 m 7-Oct-2009, leg. J. Lenz	**Molecular** BC ZSM Lep 48877: COI –BIN BOLD:ABV5494	ZSM
***Agoma trimenii*** m	Zimbabwe: Mashonaland, Great Dyke Mts., 28 km S Miombo, 350 m, 17°53.35′S 30°58.67′E, 05-Jan-2011, leg. J. Lenz	**Molecular** BC ZSM Lep 48875 COI –BIN BOLD:AAV6347	ZSM
***Heraclia africana*** f	South Africa: Natal, Umlazazi-NR, Mtunzini, 53 m, 29°58.33′S 32°25′E, 22-Mar-1997, leg. M. Ochse	**Molecular** BC ZSM Lep 47510 COI –BIN BOLD:ABW4822	ZSM
***Schausia coryndoni*** f	Zimbabwe: Manicaland, Nyanga, Vukutu, 1900 m, 18°35.11′S 32°60.58′E, 27-Jan-2011, leg. J. Lenz	**Molecular** BC ZSM Lep 48876 COI –BIN BOLD:ABV4463	ZSM
***Periscepta polysticta*** m	Australia: Queensland, 16°8′S 145°63.3′E, 1-15-Dec-2005, leg. D. C. Rentz	**Molecular** MM07669 COI –BOLD:AAM5020. See Zahiri et al. (2013) for nine other genes	UOZM
***Agarista agricola*** m	Australia: Queensland, Edungalba, 23°71.6′S 149°85.1′E, 10-Nov-1975, leg. A. W. Smith	**Molecular** 10ANIC-06923 COI –BIN BOLD:AAJ1139	ANIC

### Molecular techniques

We attempted to sequence all eight molecular markers as in Zahiri et al. (2013) for *Cartaletis dargei*, but only two gene regions amplified successfully from three specimens (collected in 2010, 2013, 2015, see Table 1), the first half of COI (the DNA barcode region) and *wingless*. In addition, one further specimen yielded the DNA barcode (collected in 2003, see Table 1). Primers and protocols were taken from Wahlberg & Wheat (2008). The successful PCR products were Sanger-sequenced by Macrogen Europe (Amsterdam). Chromatograms were checked with BioEdit (Hall, 1999), and aligned sequences were submitted to NCBI GenBank and are maintained in the VoSeq database (Peña & Malm, 2012).

### Alignment and phylogenetic analyses

The *Cartaletis dargei* sequences were initially analysed in a dataset of eight genes (CAD, COI, EF-1a, GAPDH, IDH, MDH, RpS5 and *wingless*) where all major lineages of Macroheterocera were represented (taken from Rajaei et al., 2015). Based on the results (*Cartaletis dargei* fell within Noctuidae), we analysed the new sequences in the eight gene dataset from Zahiri et al. (2013), which has an aligned concatenated length of up to 6400 bp for 78 Noctuoidea species. In addition, we included DNA barcode sequences (Hebert et al., 2003) from six species of Agaristinae from Africa and Australia (see Table 1). Further, we analysed the same taxa using up to two genes only (COI and *wingless*), which had an aligned concatenated length of up to 1,876 bp. The aim was to compare how up to two genes versus up to eight genes included in the phylogenetic analysis may affect the systematic position of *Cartaletis dargei*.

Phylogenetic analyses were carried out using IQ-TREE 1.6.10 (Nguyen, Von Haeseler & Minh, 2015) in a maximum likelihood framework. The data were partitioned by gene and analysed with the partition finding (Chernomor, von Haeseler & Minh, 2016) and model finding (Kalyaanamoorthy et al., 2017) algorithms of IQ-TREE (using the command MFP+MERGE). Multiple runs were completed, and within one run 100 independent searches were made. Robustness of the results were assessed using UFBoot2 (Hoang et al., 2018) and a SH-like approximate likelihood ratio test (Guindon et al., 2010), each with 1000 replicates. Analyses were run on the CIPRES server (Miller, Pfeiffer & Schwartz, 2010).

### Morphological analyses

Genitalia and abdomens were prepared following standard methods (for instance (Hardwick, 1950)). The male aedeagus is shown both with uneverted vesica, to allow comparison with older literature, and with everted vesica. The vesica was everted via the caecum that was cut open by placing the aedeagus inside a hypodermic syringe (Sihvonen, 2001). Some structures were photographed during dissection in situ using The Fixator (Wanke et al., 2019), to allow an optimal angle for observing and illustrating certain structures. The wings were descaled using the method described in Sihvonen (2005). All structures except wings were stained with Chlorazol Black. Numerous dissected structures shown in the plates were photographed in two to six images at different depths of focus, using a Leica DM1000 microscope and Leica DFC295 camera, and combined into single images using image-stacking software in Adobe Photoshop CC v.20.0. Larger structures were photographed using Canon EOS 5D digital camera with MP-E 65 mm EF 100 mm macro lense. Photos were taken with StackShot automated macro rail and focus stacked in Image Manager Software (Zerene Stacker). Scanning electron microscopy was performed with FEI Quanta 250 FEG (Thermo Fisher, Oregon, USA) using ETD detector of secondary electrons. As the specimen was uncoated, the low accelerating voltage of 1 kV and spot size 3 were used to minimize charging. Original images were cleaned and edited in Adobe Photoshop and compiled into plates with CorelDRAW 2020. Genitalia terminology follows Klots (1970) and Sibatini (1972), wing venation follows Wootton (1979). In ambiguous cases, descriptive terms were used and were accompanied by illustrations.

### DNA barcodes, genus- and species-level taxonomy

The genetic data, together with morphology and other available evidence such as distribution, were used to draw conclusions on the exact systematic position of *C. dargei* within Agaristinae. DNA barcodes (658 bp region near the 5′ terminus of the COI mitochondrial gene) of seven specimens (Table 1) were studied using the analytical tools on BOLD, including BIN (Barcode Index Number) and barcode gap analysis (Ratnasingham & Hebert, 2007; Ratnasingham & Hebert, 2013). Genetic divergences between sequences were calculated using the number of base differences between sequences as implemented in MEGA X (Kumar et al., 2018), and they are reported as percentages. The taxonomic and collection data, voucher image, COI sequence and other metadata are available on the BOLD database https://v4.boldsystems.org through the public dataset DS-AGARIST, doi: dx.doi.org/10.5883/DS-AGARIST “Agaristinae - dargei and imperialis”.

## Results

### Phylogenetic position of “*Cartaletis*” *dargei*

**Figure 2:**
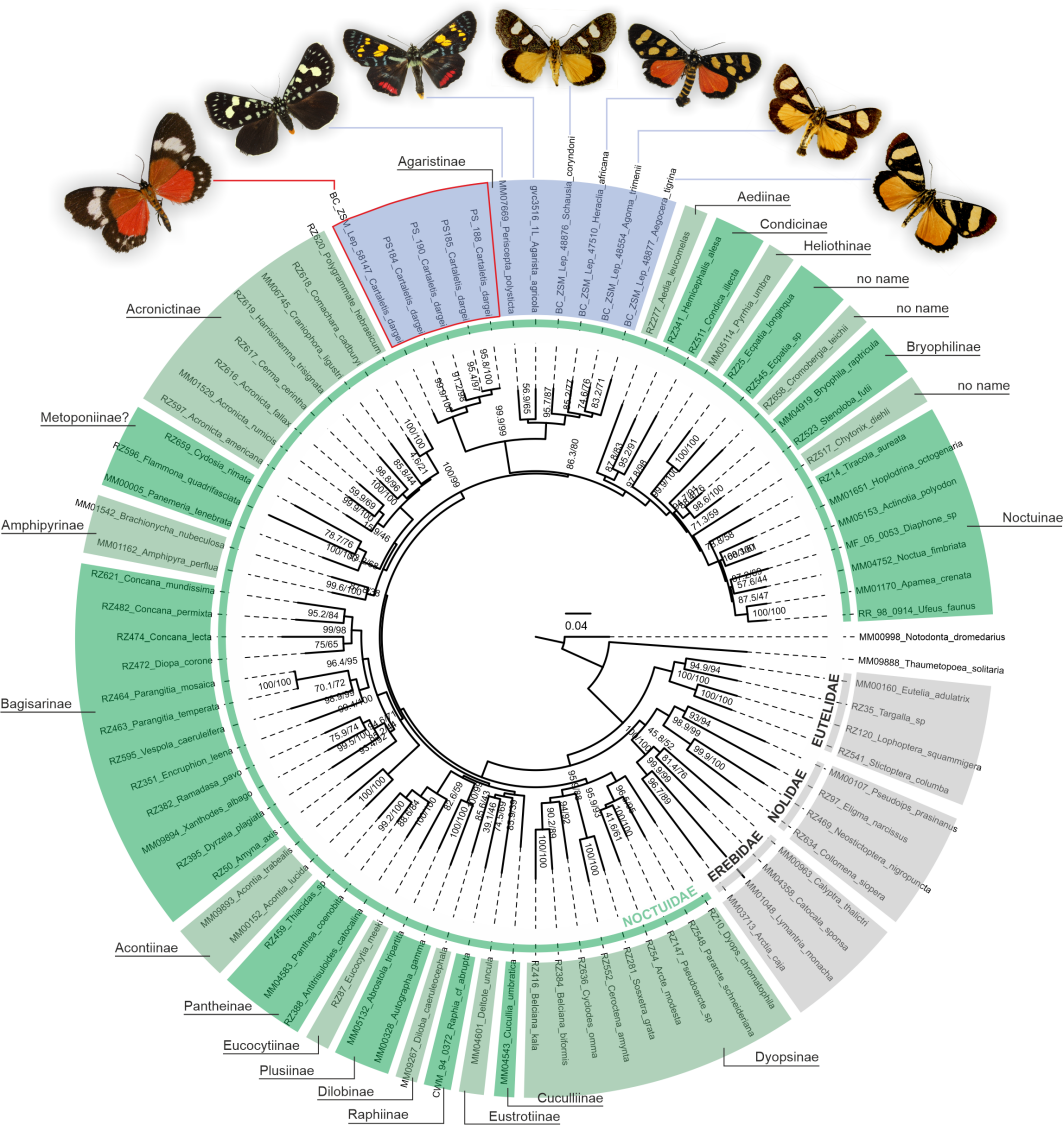
Phylogenetic relationships of Noctuidae, showing the position of “*Cartaletis” dargei*. ”*Cartaletis*” *dargei* is reclassified here as *Aletopus dargei* (Herbulot, 2003) **comb. n.** (highlighted with red) within the subfamily Agaristinae (highlighted with blue). Majority of data are from Zahiri et al. (2013), which is also followed for the subfamily classification. Numbers above branches are SH-like/UFBoot2 support values.

A phylogenetic analysis of 78 Noctuoidea species (up to eight molecular markers/species) with five *Cartaletis dargei* samples (up to two molecular markers/specimen) and six Agaristinae species (all eight molecular markers for *Periscepta*, COI barcode only for the other five species) recovered *C. dargei* in a well-supported Noctuidae: Agaristinae position (SH-like/UFBoot2 = 99.9/99) (Fig. 2). African *C. dargei* was recovered as sister to all other analysed Agaristinae. African (*Schausia coryndoni, Heraclia africana, Agoma trimenii, Aegocera tigrina*) and Australian species (*Periscepta polysticta, Agarista agricola*) grouped together, each in separate lineages. The analysis of up to two molecular markers/species (COI and *wingless*) recovered *C. dargei* in exactly the same position within Noctuidae: Agaristinae (SH-like/UFBoot2 = 100/100) (File S1).

**Figure 3:**
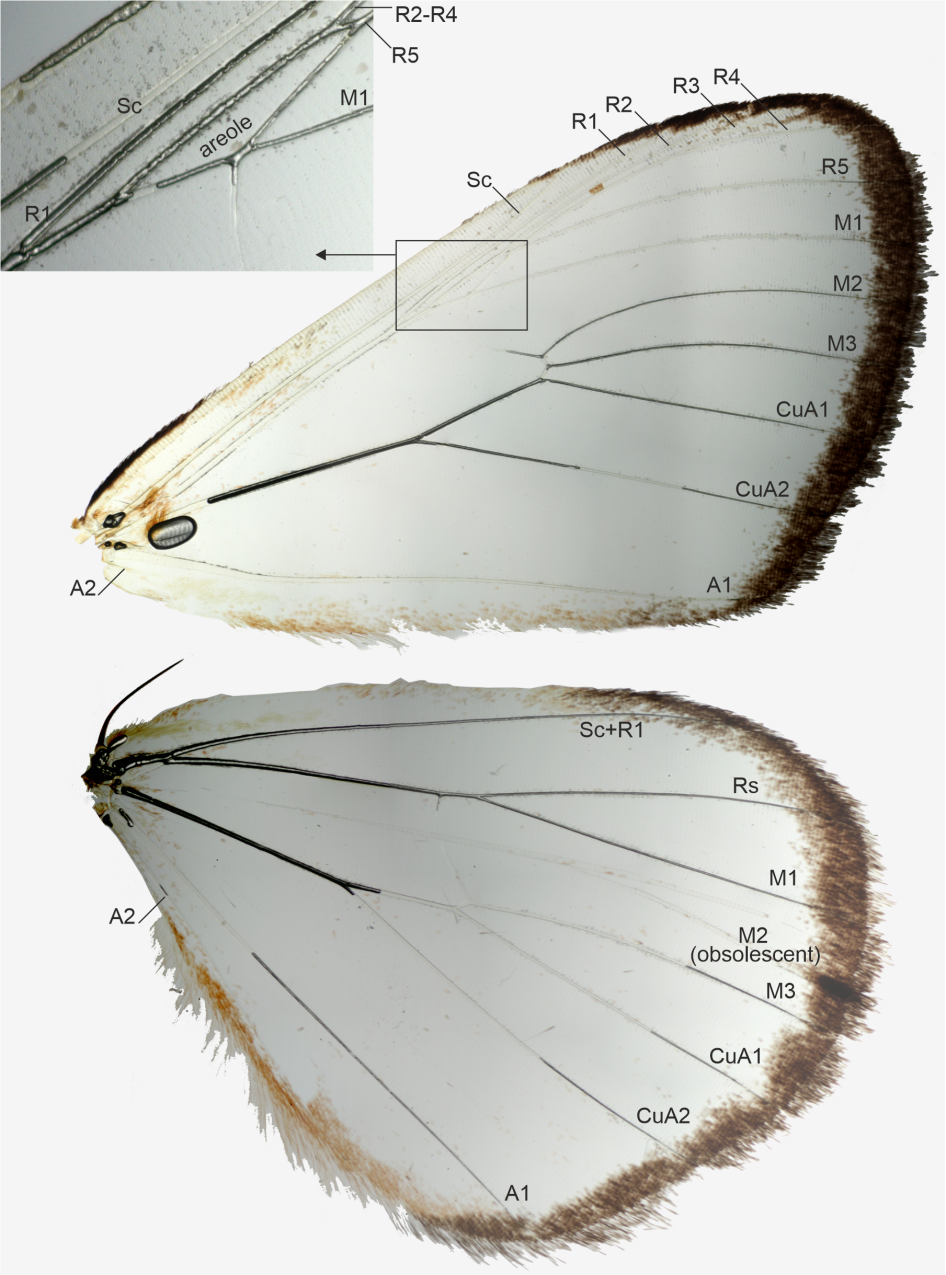
Wing venation of a male, belonging to *Aletopus dargei* group. Upper left corner shows part of the forewing enlarged. Malawi: Chitipa district, Mughese forest, 6000 ft, 9-16 Jan. 2002 (coll. HSS, Sihvonen dissection 2047).

**Figure 4:**
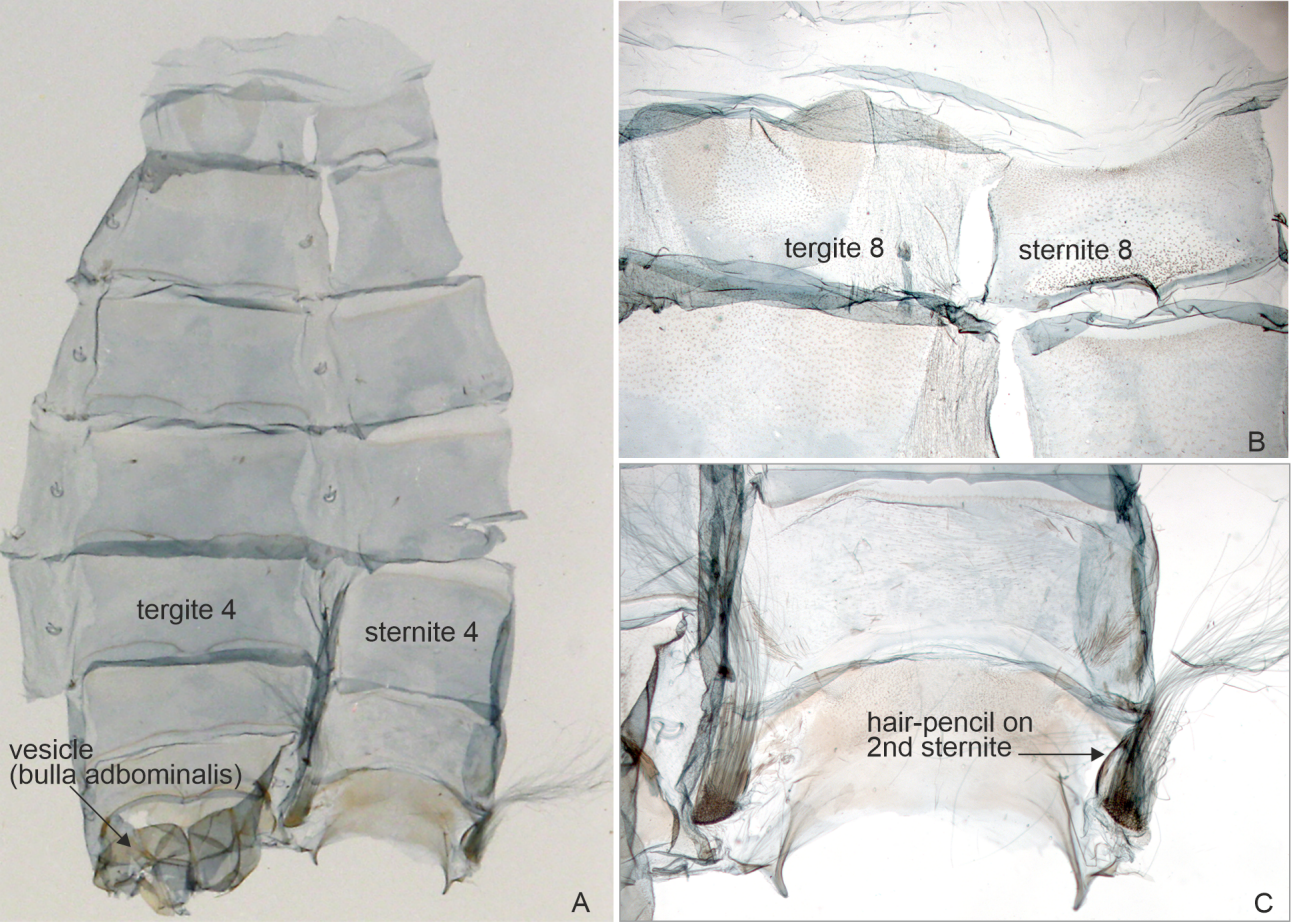
Abdominal structures of *Aletopus* male. (A) Overview. (B) Segment A8 enlarged. (C) Sternite A2 enlarged. Male abdominal structures are similar in all examined *Aletopus* material. Tanzania: West Usambara, Magamba Forest, 2000 m., 04.43.399 S - 038.14.744 E, Dec. 2003, leg. Ph. Darge (coll. HSS, Sihvonen dissection 2078).

**Figure 5:**
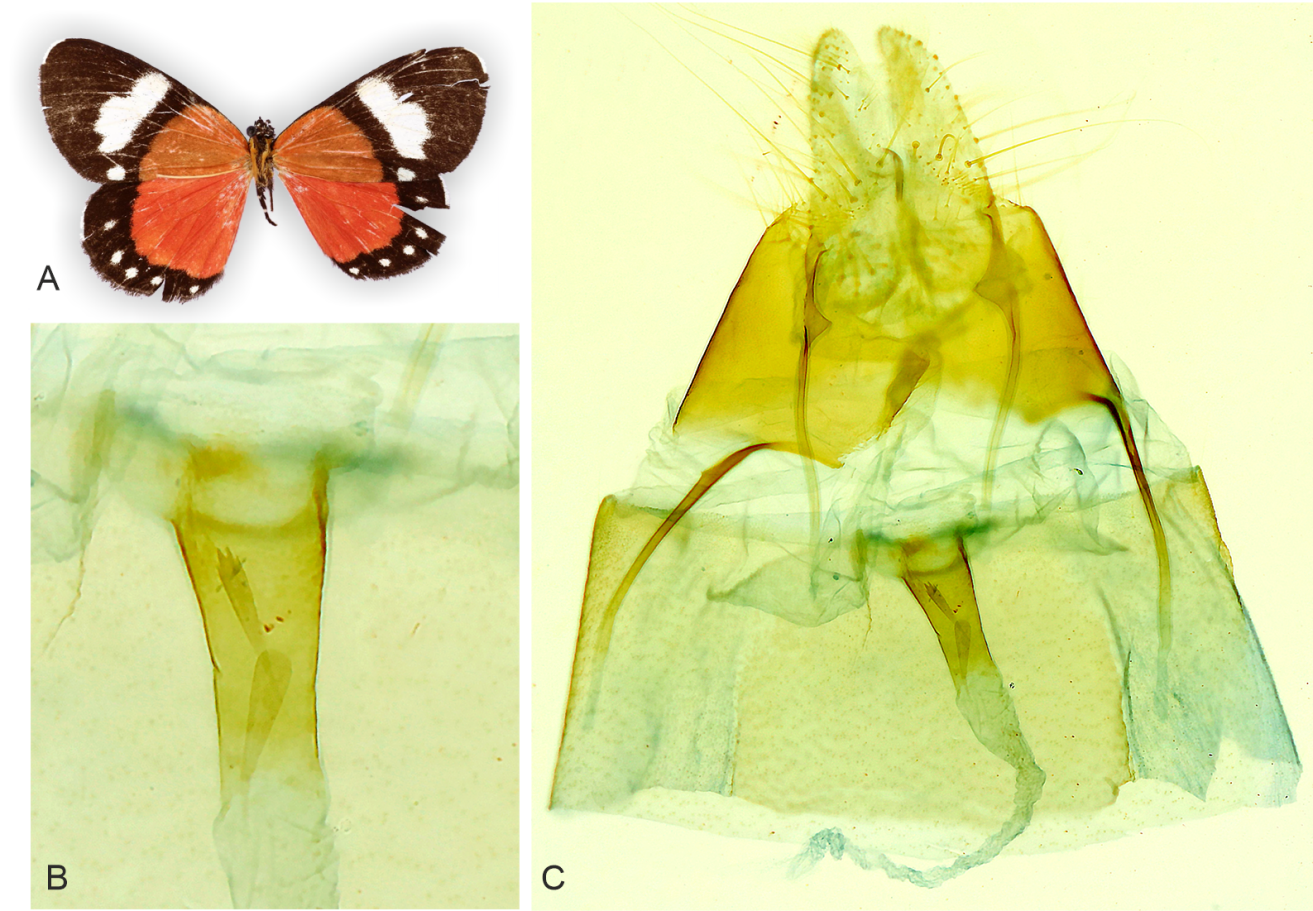
Holotype adult and genitalia of *Aletopus imperialis*Jordan, 1926. (A) Female adult. (B) Enlarged ostium bursae and adjacent structures. (C) Female genitalia. Corpus bursae is missing in the holotype (dissection artefact). Tanzania: Tanganyika territory, Bungu, Usumbara (coll. NHMUK, Noctuidae Brit. Mus. slide No. 8298/Maureen Lane).

**Figure 6:**
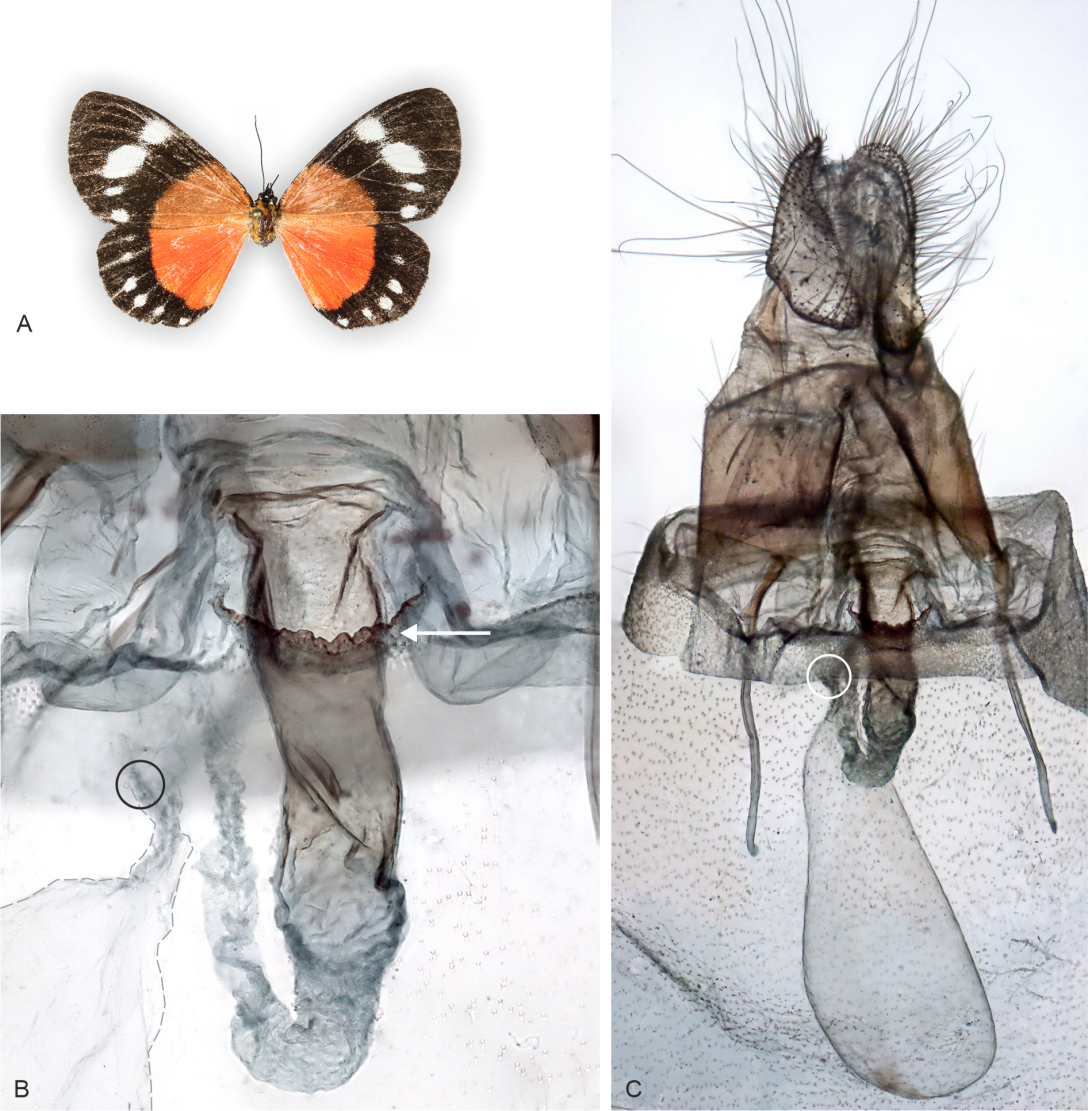
Adult and genitalia of *Aletopus*, belonging to the *Aletopus dargei* group. (A) Adult. (B) Enlarged ostium bursae and adjacent structures. (C) Female genitalia. Arrow indicates the rough margin of the ostium bursae, compare it against the smooth margin in the holotype of *A. imperialis* shown in Fig. 5. Circle indicates the point of origin of the ductus seminalis. Margins of corpus bursae partly highlighted in Fig. 6B. Fig. 6C was photographed in ethanol during dissection to show the membranous structures in full expanse. Tanzania: Milo, 1,800 m., 10°00′30″S–34°38′09″E, 4 Mar. 2013, leg. ABRI MH-PW (coll. HSS, Sihvonen dissection 2052).

**Figure 7:**
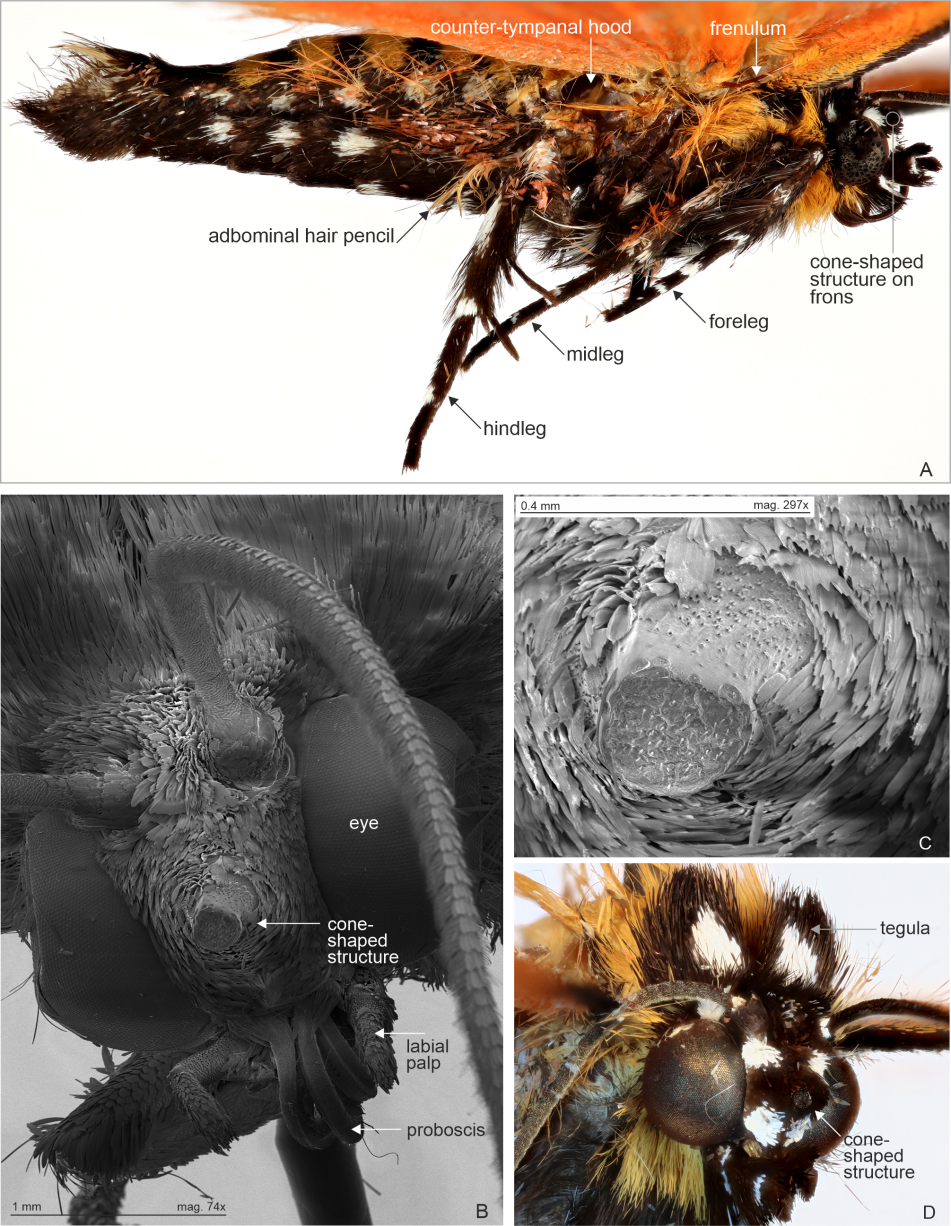
Male abdomen in lateral view and head with cone-shaped protuberance, specimen belonging to *Aletopus dargei* group. Abdominal hair pencil (present in males) and cone-shaped structure on frons (present on both sexes) are diagnostic Agaristinae structures. Few other characters are indicated for orientation. (A) Male abdomen in lateral view. Malawi: Chitipa district, Mughese forest reserve, 6000 ft, 9–16 January 2002 (coll. HSS). (B) Head with cone-shaped structure, SEM photograph. Malawi: Chitipa district, Mughese forest, 6000 ft, 9–16 January 2002 (coll. HSS). Photo by Ilya Belevich, used with permission. (C) Detail of cone-shaped structure, SEM photograph. Malawi: Chitipa district, Mughese forest, 6000 ft, 9–16 January 2002 (coll. HSS). Photo by Ilya Belevich, used with permission. (D) Head with cone-shaped structure, normal photograph. Tanzania: West Usambara, Magamba forest, 2,015 m, December 2003 (coll. ZSM).

Morphology supports a position within Agaristinae also: wing venation trifine (Fig. 3); hindwing vein M2 obsolescent (Fig. 3); male with trifine brush-organs complete of levers, hair-pencils and pleuro-sternal pockets (Fig. 4); counter-tympanal membrane greatly enlarged and associated with reduced counter-tympanal hood (not figured); paired vesicle (bulla abdominalis) on A1 (Fig. 4); female without signum on corpus bursae (Figs. 5–6); forewing often with pale spots or bands on black background (characters after Holloway, 2001); cone-shaped prominence on frons of head (Fig. 7) (character after Becker, 2010). Several Agaristinae species have white fringes on forewing apex, which is also present in our study species. Larva and pupa of *Cartaletis dargei* are unknown, therefore the diagnostic characters (for instance Kitching & Rawlins, 1999 and references therein) could not be evaluated. We illustrate the characteristic cone-shaped prominence on the frons of the head (Fig. 7), which we have found across Agaristinae studied from Australia, Thailand, Africa, and which is mentioned by Becker (2010) to occur in the Neotropical species. The apex is crater-shaped with tiny sensilla (Fig. 7). See Discussion for additional information.

After the Agaristinae position of *Cartaletis dargei* was established, an extensive screening of Agaristinae materials in collections and examination of literature revealed that taxon *dargei* should be classified in the monotypic genus *Aletopus* (Jordan, 1926). The genus combination was supported by DNA barcodes and morphology, but the species-level taxonomy turned out to be complex. Because *Aletopus* has not been subject to taxonomic revision, we decided to provide such. Taxonomic conclusions are presented below.

### Taxonomy of genus *Aletopus* (Lepidoptera, Noctuidae: Agaristinae)

**Table:** 

***Aletopus*Jordan, 1926**
*Aletopus*Jordan, 1926, Novitates Zoologicae 33: 376. Type species: *Aletopus imperialis*Jordan, 1926, by original designation.

*Aletopus,* as reclassified here, contains three described species, but it could contain five or even more species. We had access to seven DNA barcoded specimens, whose morphology was studied also, in addition to other material (Table 1). Both genetic and morphological data are compatible with a number of taxonomic scenarios, but because material on both sexes was rather limited, we took a conservative view and formally recognise with certainty only two species. See Discussion for alternative classifications.

**Table:** 

***Aletopus imperialis*Jordan, 1926***Aletopus imperialis* (Jordan, 1926). Novitates Zoologicae 33: 377, Fig. 1, [Tanzania]: Tanganyika territory, Bungu, Usumbara. Holotype: female (in NHMUK). Examined, including genitalia (Noctuidae Brit. Mus. slide No. 8298, prep. by Maureen Lane (maiden name Grogan))

**Figure 8:**
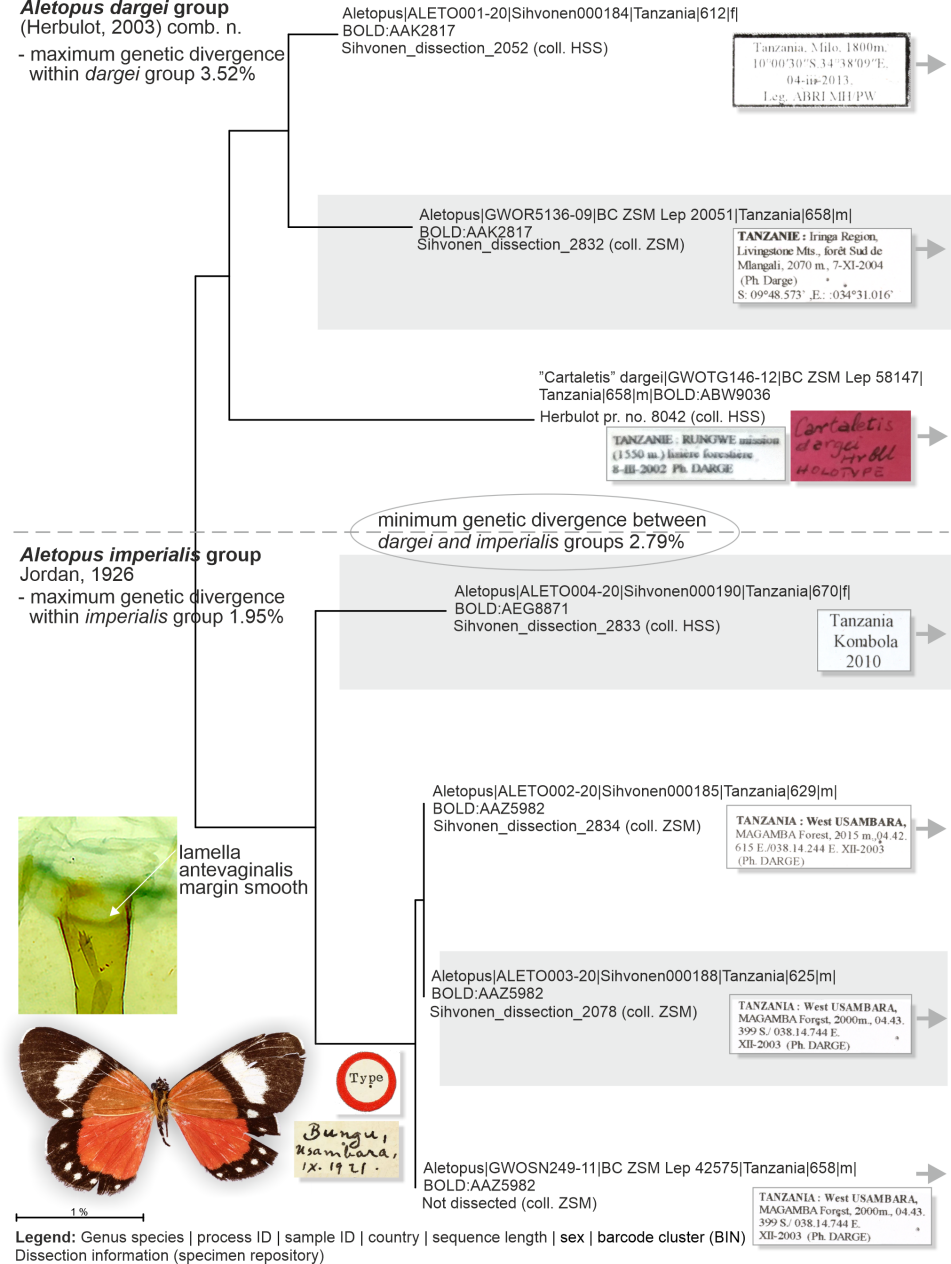
Neighbor-joining tree based on the barcode fragment of the COI gene in genus *Aletopus* (part 1/2 of Figure). Holotype of *A. imperialis* was not barcoded, but morphology supports its association with the specimens on lower part of tree.

**Figure 9:**
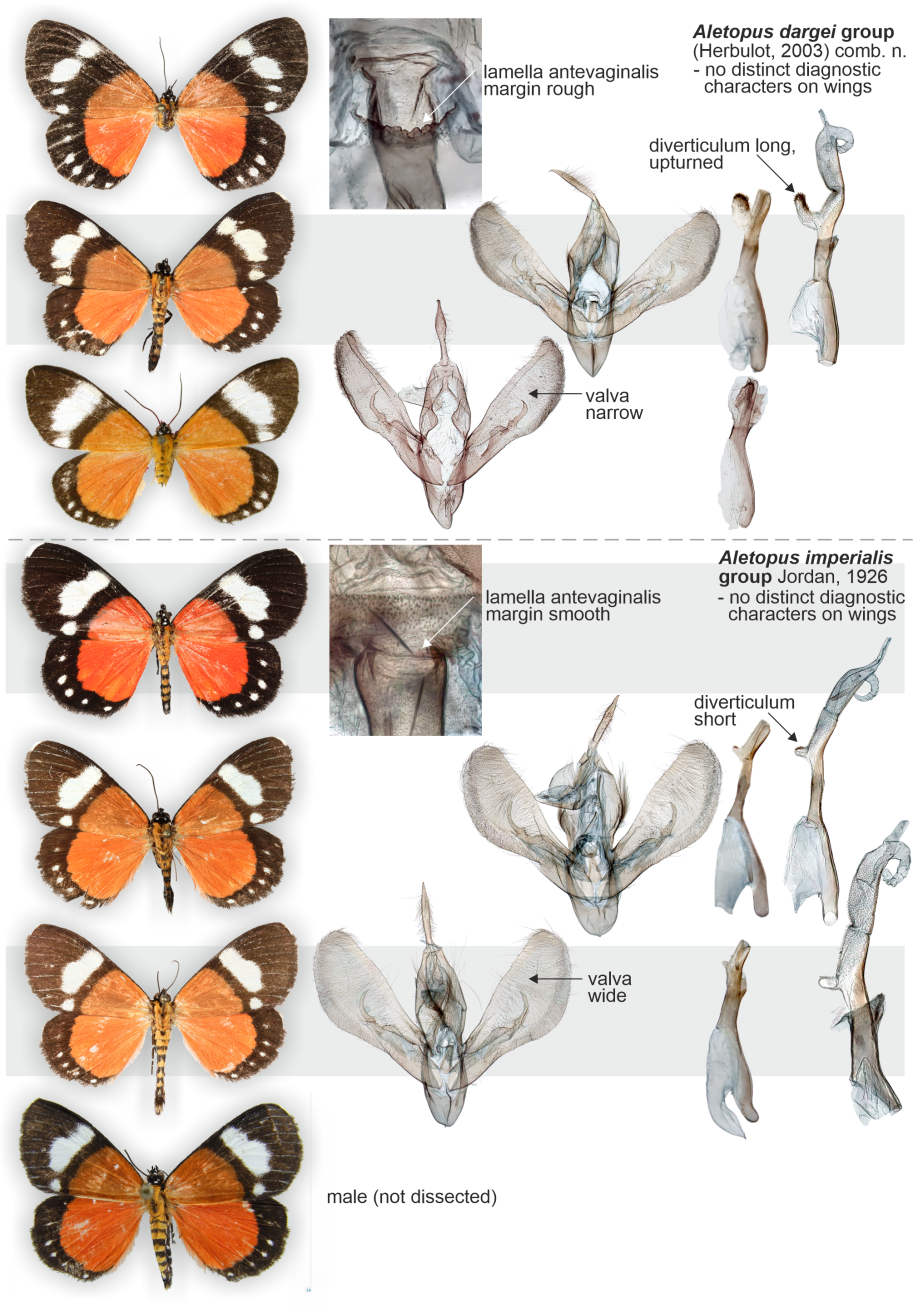
Barcoded specimens and selected morphological structures (part 2/2 of Figure). More male variation is shown on Fig. 13.

**External characters and abdomen** (Figs. 5, 8 and 9): Wingspan: males 36–42 mm (*n* = 7), females 42–44 mm (*n* = 2). Wings rounded. Basal part of wings orange to red, distal part blackish brown, traversed by white band or spots on forewings, by row of small white dots on hindwings. Terminal line black, except apex white on both wings. Wings below as above. Frons with four lateral white spots, medial area black with cone-shaped projection. Eyes large, lined ventrally by long and narrow yellow or orange scales. Proboscis well-developed. Basal parts of labial palps white, otherwise structures black. Medial parts of tegulae white, otherwise structures black. Antenna filiform in both sexes. Mesothorax behind tegulae with long and narrow yellow or orange scales. Legs mostly black, with white scales on proximal parts of segments. Spur formula 2–2–4 in both sexes. Abdomen slender, dorsally orange-brown banded; laterally black with two rows of white dots; ventrally black with one row of white dots on segments 1–3. Counter-tympanal membrane greatly enlarged, associated with reduced counter-tympanal hood. Paired vesicle (bulla abdominalis) on A1. Male abdomen with trifine brush-organs on A2 and tergite A8 weakly horse-shoe shaped (Fig. 4), other abdominal sternites and tergites of both sexes undifferentiated. **Variation**: *Aletopus imperialis* is slightly sexually dimorphic; the males are orange, while the females show redder colouration, particularly on the hindwings. Reddish-orange males do exist, but normally the colour is not as deep as in the females. Basal part of the female forewing can also be slightly reddish-brown. Occasionally, veins are weakly visible as narrow white lines on black areas.

**Venation** (Fig. 3): Typical trifine venation of Noctuidae. Forewing R veins form an elongated areole. Hindwing vein M2 obsolescent. Both wings with one anal vein (1A + 2A), A2 short and reduced. Jordan (1926, Fig. 1) illustrates part of the hindwing venation, matching with the venation illustrated here.

**Male genitalia** (Fig. 9, lower part): Uncus long, setose, expanded medially. Tegumen long and narrow, ventral margins slightly triangular, covered with long setae, dorsal sclerotisations very narrow, y-shaped. Valva long, narrow at base, widest subapically, ventral margin weakly concave, apical half covered with setae. Valva with hook-shaped process (harpe) and sclerotised ridge in middle (sacculus). Saccus narrow, elongated. Aedeagus long, narrow, curved ventrally, dorsal part of shaft broadly open at junction with ductus ejaculatorius for about half of aedeagus length, caecum short. Basal extension of vesica short, blunt-ended, covered with minute spines. Vesica long, tubular, covered with microcornuti, with ventrally recurved tip after ductus ejaculatorius.

**Figure 10:**
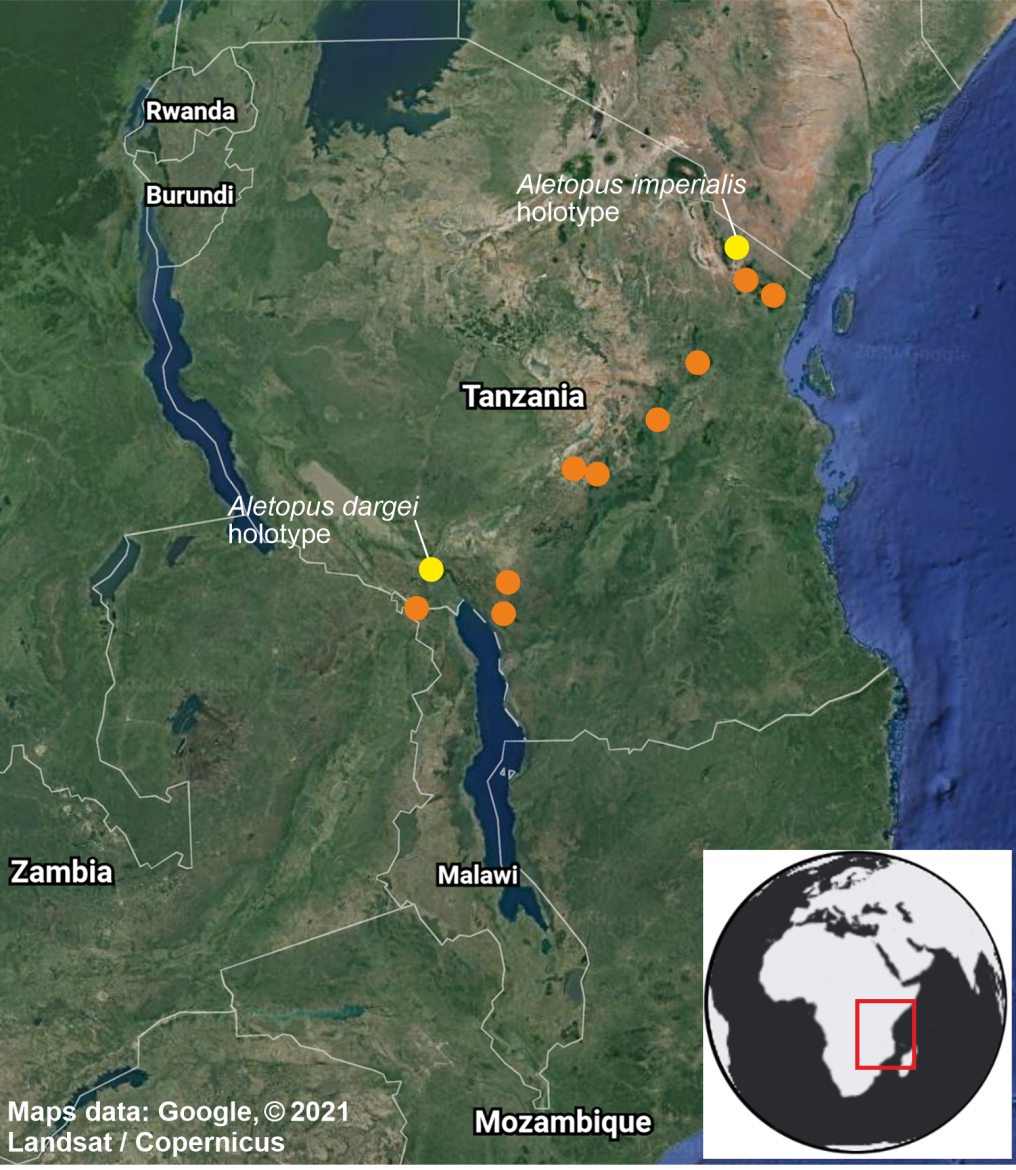
Distribution of *Aletopus* in Tanzania and Malawi, based on the samples examined in this study. Type localities of *A. imperialis* and *A. dargei* **comb. n.** are highlighted with yellow. Map data ©2021 Google.

**Female genitalia** (Fig. 5): Papillae anales prominent, covered with long setae. Apophyses posteriores slightly shorter than apophyses anteriores. 8th segment with membranous pouches laterally, on both sides of ostium bursae. Ventral margin of ostium bursae smooth. Lamella postvaginalis membranous. Ductus bursae narrow, sclerotised. Holotype female (Noctuidae Brit. Mus. slide No. 8298 lacks corpus bursae (dissection artefact), therefore we exclude description of the corpus bursae (see. *A. dargei*).

**Table 2:** Minimum Kimura 2-parameter divergences (%) among specimens in the *Aletopus imperialis* and *dargei* species groups. Divergences are based on the analysis of the DNA barcode sequence of the COI gene. Each analysed specimen has sample ID and BIN number. Figures 8–9 show the graphical presentation of divergences and morphology of the associated specimens.

	Sihvonen190— BOLD:AEG8871 *imperialis* group	Sihvonen188— BOLD:AAZ5982 *imperialis* group	Sihvonen185— BOLD:AAZ5982 *imperialis* group	Sihvonen184— BOLD:AAK2817 *dargei* group	BC_ZSM_Lep_42575— BOLD:AAZ5982 *imperialis* group	BC_ZSM_Lep_20051— BOLD:AAK2817 *dargei* group	BC_ZSM_Lep_58147— BOLD:ABW9036 *dargei* group
Sihvonen190— BOLD:AEG8871 *imperialis* group	0						
Sihvonen188— BOLD:AAZ5982 *imperialis* group	1,95	0					
Sihvonen185— BOLD:AAZ5982 *imperialis* group	1,91	0,00	0				
Sihvonen184— BOLD:AAK2817 *dargei* group	2,96	2,79	2,79	0			
BC_ZSM_Lep_42575— BOLD:AAZ5982 *imperialis* group	1,83	0,00	0,00	2,85	0		
BC_ZSM_Lep_20051— BOLD:AAK2817 *dargei* group	3,67	3,65	3,58	1,34	3,36	0	
BC_ZSM_Lep_58147— BOLD:ABW9036 *dargei* group	4,43	4,48	4,39	3,52	4,13	3,36	0

**Distribution, habitat, phenology, biology**: Exact distribution is not known, requiring dissection and DNA barcoding of additional material. Therefore the records on the distribution map are not more detailed as shown. *Aletopus* species are known from eastern Africa, from Tanzania to Malawi (Fig. 10). Most records are from forest habitats between 1800–2070 m, one specimen is from 418 m. The specimens have been recorded between September and March, most specimens are from December, potentially hinting to one generation per year. It is uncertain whether *Aletopus* species are nocturnal or diurnal. The material in coll. ZSM was collected by Philippe Darge, and the specimens were among artificial light-collected material when those arrived to museum, therefore indicative of being collected at light. The material in coll. HSS was collected during day as bycatch by butterfly collectors, who were doing fieldwork for the African Butterfly Research Institute (ABRI, Kenya). Otherwise, biology and immature stages are unknown.

**Genetic data** (Fig. 8, Table 2): *Aletopus imperialis* group splits between two Barcode Index Numbers (BINs): BOLD:AAZ5982 is represented by three specimens from Tanzania (barcode length 625–658 bp), and of those three, BC_ZSM_Lep_42575 is the nearest to the BIN BOLD:AEG8871 at minimum pairwise distance of 1.83%.

**Figure 11:**
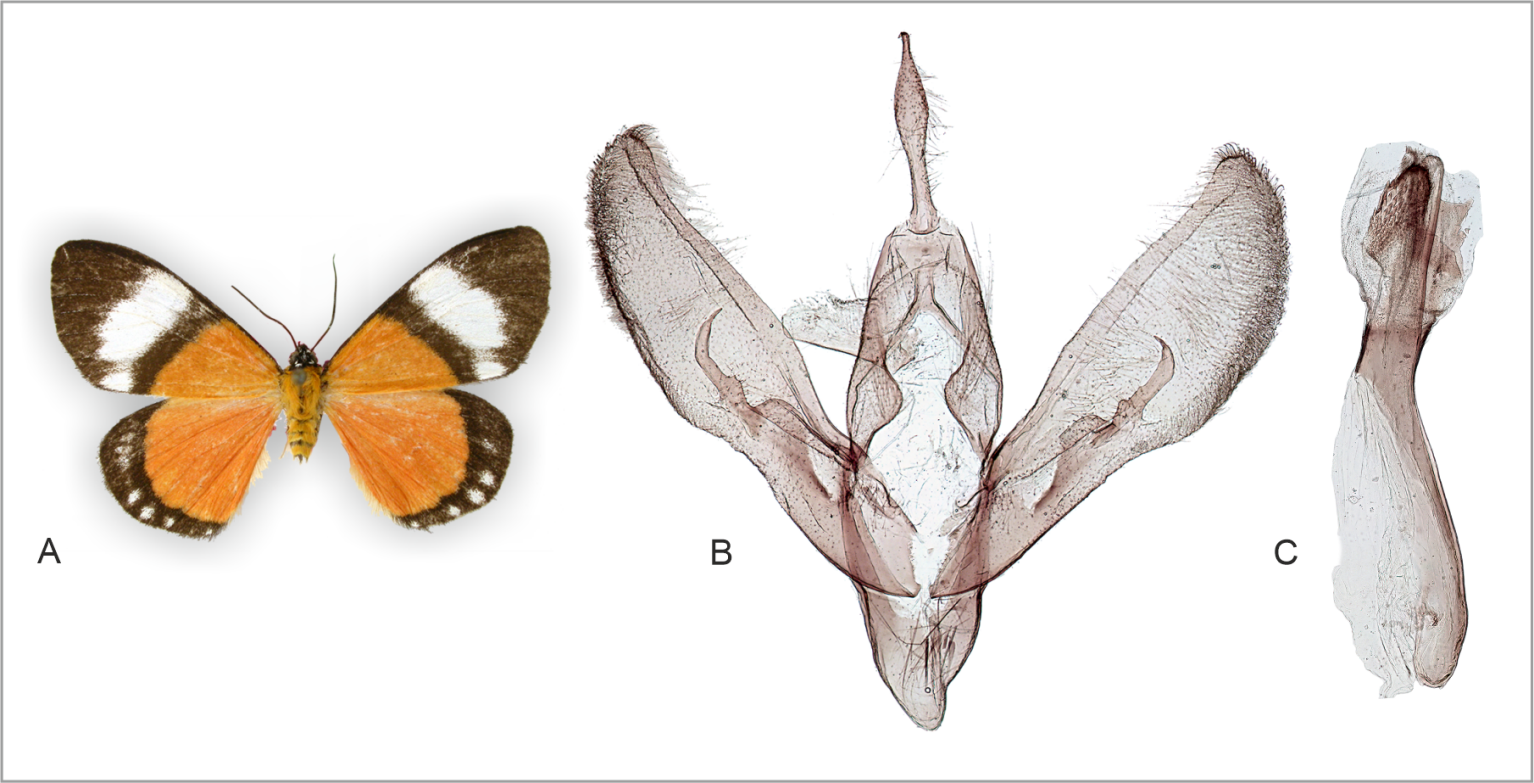
Holotype adult and genitalia of “*Cartaletis*” *dargei*, which is reclassified here as *Aletopus dargei* (Herbulot, 2003) comb. n. (A) Male adult. (B) Male genitalia. (C) Aedeagus. Dissected abdomen of the holotype is not complete (dissection artefact), therefore it is not illustrated, see Fig. 4. Tanzania: Rungwe mission, Iisière forest, 1550 m, 8 Nov. 2002 (coll. ZSM, dissection 8042/Herbulot, barcoded ZSM Lep 58147).

**Figure 12:**
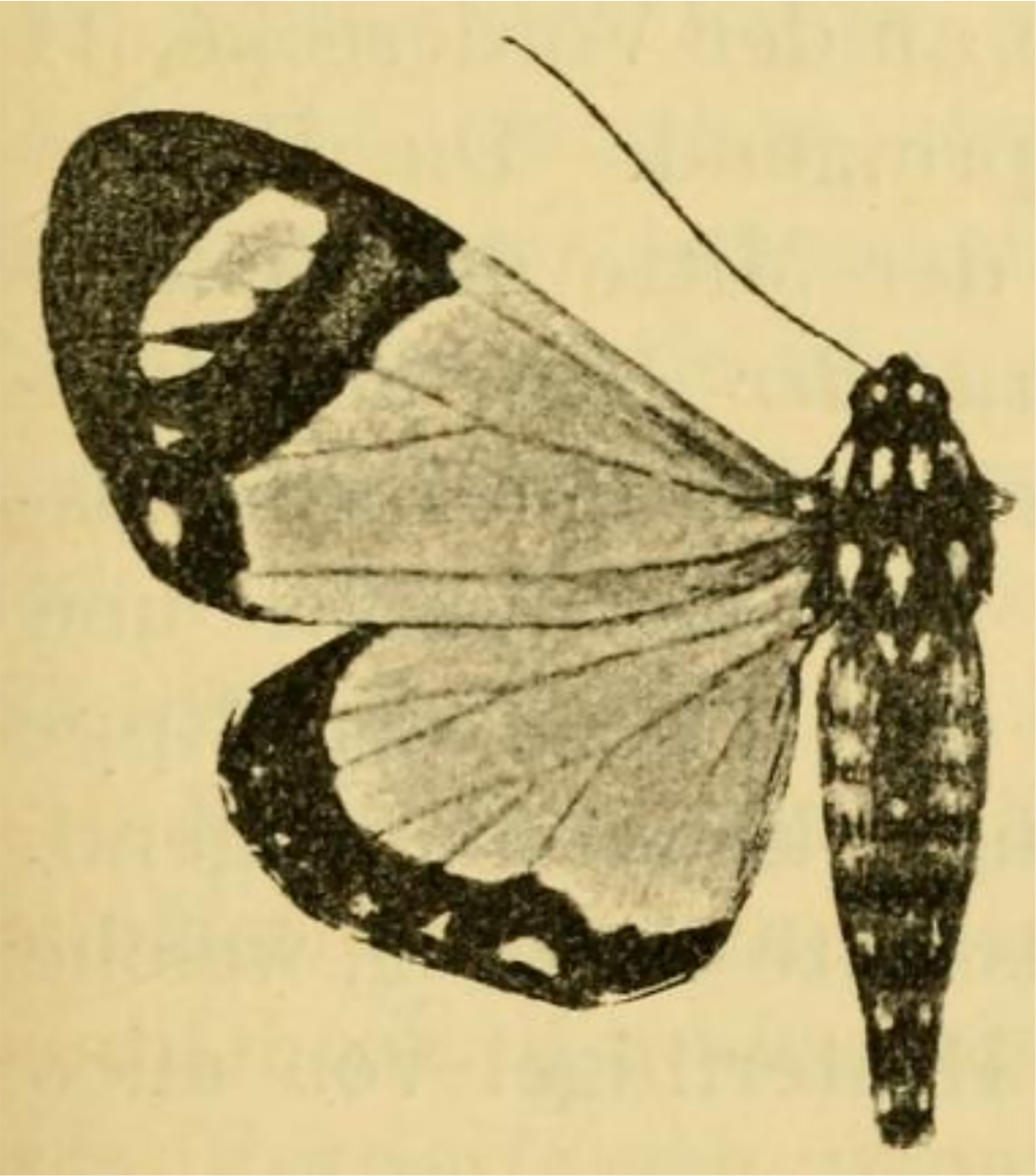
Holotype of *Aletopus ruspina* (Aurivillius, 1909) from Republic of the Congo. The identity and generic combination of the species are uncertain, because the type is lost. Reproduced from the original publication (Aurivillius, 1909, Fig. 43), available on Biodiversity Heritage Library https://www.biodiversitylibrary.org/page/6393000.

**Similar species**: *Aletopus imperialis* and *A. dargei* are externally very similar, and so far reliable external diagnostic characters have not been found. Basal extension of vesica is short and straight in *A. imperialis* (digitiform and curved in *A. dargei*), see Figs. 8 and 9. Margin of lamella antevaginalis is smooth in *A. imperialis* (rough in *A. dargei*), see Fig. 5–Figs. 6 and 8, Fig. 9. Minimum pairwise distance between *A. imperialis* group and *A. dargei* group is 2.79% (Figs. 8 and 9, Table 2). In addition, *Aletopus* taxa are superficially similar to unrelated *Euphaedra ruspina* (Hewitson, 1865) (Nymphalidae), which occurs sympatrically in eastern Africa, and to a lesser degree to *Apaegocera aurantipennis* Hampson, 1912 (Agaristinae).

**Table:** 

***Aletopus dargei*Herbulot, 2003 comb. n***Cartaletis dargei*Herbulot, 2003. Lambillionea 103: 126, Figs. 6 and 13. Tanzania: Rungwe Mission, Iisière forestière, 1550 m. Holotype: male (in ZSM). Examined, including genitalia (slide Pr. No. ZSM G [1]8042, prep. by Claude Herbulot). Transferred here from Geometridae: Sterrhinae to Noctuidae: Agaristinae **comb. n.**, based on molecular and morphological data.

**Figure 13:**
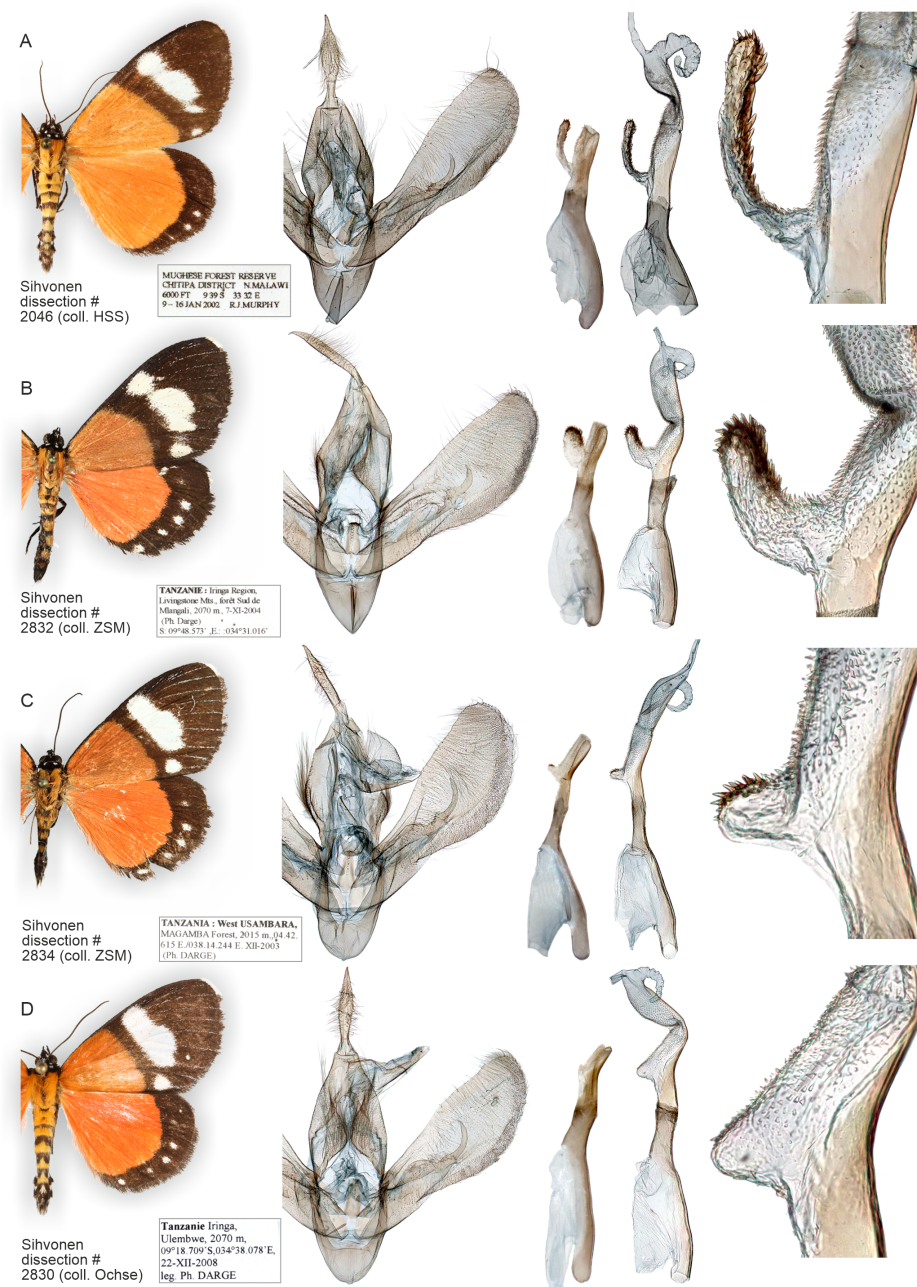
Variation of wings and male genitalia in genus *Aletopus*. The most variable structures are the shape of uncus, valva and the diverticulum of vesica (shown enlarged on the right margin).

**External characters, abdomen, venation** (Figs. 1, 8, 9 and 11). Similar to *A. imperialis*, so far reliable external diagnostic characters have not been found. See *A. imperialis* for a general description, including venation.

**Male genitalia** (Fig. 11): Similar to *A. imperialis*, but with following differences. Basal extension of vesica is digitiform and curved in *A. dargei* (short and straight in *A. imperialis*). Also, valva appears narrower in *A. dargei*, and wider in *A. imperialis*, but this quantitative character must be used with caution, because the width may appear different if viewed from different angles.

**Female genitalia** (Fig. 6): Similar to *A. imperialis*, but with following difference. Margin of lamella antevaginalis is rough in *A. dargei* (smooth in *A. imperialis*). Ductus bursae is distinctly curved before opening of ductus seminalis. Corpus bursae large, elongated, very thin, with short sub-conical appendix bursae posteriorly from which ductus seminalis arises. Signum absent.

**Distribution, habitat, phenology, biology**: See text under *A. imperialis*.

**Genetic data** (Fig. 8, Table 2): *Aletopus dargei* group splits between two Barcode Index Numbers (BINs): BOLD:ABW9036 is a singleton (658 bp) from Tanzania (holotype of *A. dargei*), and the nearest BIN is BOLD:AAK2817 at minimum pairwise distance of 3.4%. BOLD:AAK2817 (in *dargei* group) is represented by two specimens (612–658 bp) from Tanzania, and the nearest BIN is BOLD:AAZ5982 (in *imperialis* group) at minimum pairwise distance of 2.8%. The complex genetic patterns of *A. dargei* and *A. imperialis* merit further attention and integrative taxonomic study (see Discussion).

**Similar species**: *Aletopus imperialis*, see text under *A. imperialis*.

**Table:** 

***Aletopus ruspina*Aurivillius, 1909** (provisional position) *Tuerta (Misa) ruspina*Aurivillius, 1909. Arkiv för zoologi 5: 9, Fig. 43. French Congo: Bonga. Holotype: male (in “Museum Bruxelles” (Royal Belgian Institute of Natural Sciences), lost. *Tuerta ruspina* was combined with genus *Aletopus* in Poole (1989).

The identity and generic combination of *Aletopus ruspina* (Aurivillius, 1909), from Republic of the Congo are uncertain. Aurivillius (1909) classified *T. ruspina* in Agaristidae and according to the original publication, the type is in “Museum Bruxelles” (Royal Belgian Institute of Natural Sciences), but it is lost according to Kiriakoff (1977). Therefore we reproduce the original illustration in Fig. 12. Based on the original illustrations, *T. ruspina* (Aurivillius, 1909, Fig. 43) and *Weymeria athene* (Weymer, 1892) an agaristine moth from Tanzania (Weymer, 1892; Goff, 2020), are similar. Aurivillius (1909) also mentions the similarity, but excludes the *ruspina-athene* relationship because *ruspina* has thorny hindleg tarsus (long bristles in *W. athene*). These both resemble in facies more *Aletis* than *Aletopus*, by being significantly larger (wingspan is over 60 mm), and the angled inner margin on the forewing blackish area near tornus is distinct, being straighter in *Aletopus*. However, the combination of *A. ruspina* and *W. athene* with *Aletis* is excluded because both these taxa have filiform antennae (bipectinate in *Aletis*). This is under the assumption the filiform antenna of *T. ruspina* are correctly depicted in the original drawing (Aurivillius, 1909, Fig. 43).

## Discussion

Agaristinae moths are predominantly diurnal (Kitching & Rawlins, 1999), with the wing facies often being bold, with pale or even metallic spots or bands on a black ground on the forewing, and often yellow, red or orange flash colourations on the otherwise black hindwing. Some species are aposematic, including the larvae, or even mimics, and the antennae may even be clubbed as in Papilionoidea (Kitching & Rawlins, 1999; Holloway, Kibby & Peggie, 2001; Braby, 2011; Lees & Zilli, 2020; Staude et al., 2020). Their colourful appearance is present even in their vernacular names; for instance, the Australian *Agarista agricola* Donovan, 1805 is called the Joseph’s coat moth or the Rainbow moth. This unusual external appearance has led to several misclassifications. One of the rarest “butterflies” (Hesperiidae) ever, which is known by a single specimen, was shown to be an agaristine moth (Zilli & Grishin, 2019) and numerous misplaced Neotropical taxa were recently transferred to Agaristinae (Becker, 2010). These moths were transferred from Erebidae: Arctiinae, Erebidae: Calpinae (=Ophiderinae), Erebidae: Pericopinae and Noctuidae: Amphipyrinae. “*Cartaletis dargei”* is another example in the sequence of misplaced Agaristinae, and we provide evidence that *Aletopus* species had been classified in two different Lepidoptera families.

Little is known about the biology of *Aletopus* species, except that specimens have been collected at rather high elevation (1800–2070 m) and based on the label data, in forest habitats. Some specimens were collected by day, thus making *Aletopus* diurnal, but it may be active at night as also explained under *A. imperialis*. Globally, more Agaristinae species live on Vitaceae than on other plant families. The same holds in the Afrotropical region, where most host records are from Vitaceae, and to lesser extent on Rubiaceae, Malvaceae and Proteaceae (Rabenstein & Speidel, 1995 and references therein; Staude et al., 2020).

We did not study the reasons causing the superficial resemblance between unrelated Lepidoptera in eastern Africa, such as *Aletopus*, *Aletis*, *Pseudaletis* (Lycaenidae) and the numerous examples in Staude & Curle (1997), but it is worth raising few points on this for future research. Staude & Curle (1997) assigned these Lepidoptera to the “wing-tip signal” assemblage. The group includes species having light spots or a band towards the apex of the forewing on a dark background, causing a flashing at the end of each clap of the flying process. It would be of particular interest to study whether the light spots or band on forewing are UV-reflective. Superficially similar white patch occurs in the Neotropical ‘clear wing complex’, which is a mimicry ring dominated by unpalatable glass wing butterflies (Nymphalidae: Danainae, Ithomiini) (Beccaloni, 1997). The white patch on the involved butterflies and moths in the Neotropics is UV-reflective, and the effectiveness of the signal has been studied using birds as predators (Corral-Lopez et al., 2020). It is unknown which predators react to this signal in Africa, but Staude & Curle (1997) speculate that the “wing-tip signal” is probably a result of the impact of the local guild of predators. If correct, this would mean that migratory intercontinental birds are unlikely predators responsible for the entrenchment of this signal. Further, according to Staude & Curle (1997) species belonging to Erebidae: Arctiinae and Lymantriinae, Noctuidae: Agaristinae, and Geometridae are assumed to be the models and *Euphaedra ruspina* (Nymphalidae) would seem to be the mimic. In addition to the specific points above, to understand even the general mechanisms of this fascinating African mimicry complex would require placing the involved taxa and lineages in a phylogenetic context, and to study their biogeographic relationships and timing of divergence.

The subfamily Agaristinae is diagnosable on the basis of morphological characters (e.g., Kitching & Rawlins, 1999; Holloway, Kibby& Peggie, 2001, see Results). An additional character was mentioned by Becker, 2010 to occur in the Neotropical species, namely the cone-shaped prominence on the frons of the head. We report this structure from African *Aletopus* also, and additionally found it across Agaristinae studied from Australia, Thailand and Africa. We therefore provide evidence it being another diagnostic character for Agaristinae, and provide detailed photographs of the structure for the first time (Fig. 7). A similar structure, which may differ in details, is present also in *Dysmilichia* Speiser, 1902, currently classified in Noctuidae: Condicinae (Hampson, 1909). We have not screened the presence of the structure more widely in Noctuidae, but somewhat similar structures are also present in some *Mudaria* Moore, 1893 (Pellinen, Mutanen & Sihvonen, 2018). Further Noctuidae examples include *Cardepia* Hampson, 1905*, Conicofrontia* Hampson, 1902*, Grotella* Harvey, 1875*, Aedophron* Lederer, 1857 and a toothed protuberance is present in Noctuoidea: Notodontidae (Basso et al., 2016). The structure is usually found in groups pupating in dry soils, assumedly enabling emerging adults to dig themselves out of hardened soils.

How many species are there in the genus *Aletopus*? The available data are limited and complicated, and several alternative taxonomic conclusions could be justified. We took a conservative approach and sorted out the material between two species, each being part of a potentially larger species group. Further, the identity and systematic position of *A. ruspina* are uncertain. To make the case transparent and to facilitate subsequent research, we present the observed morphological and genetic variation in the genus, but without further taxonomically sub-structuring this (Figs. 5, 6, 8 and 9, 13). We anticipate that new taxa that may preliminarily be inferred will need to be validated at species or subspecies level when more material on both sexes is studied, including at least morphology and DNA barcodes, in addition to relative life histories. More extensive materials are also important to assess the extent of intraspecific variation. Alternative arrangements could have been, for instance, to consider *A. imperialis* and *A. dargei* as one species and treat therefore their names as synonyms, or at the other end, to recognise up to four species in the complex. We justify our two-species hypothesis by both groupings having diagnostic morphological characters that correlate with genetic divergences (Figs. 8 and 9). If we had recognised one species only, the maximum genetic divergence (COI 5′ barcode region) within the entire group would be 4.5%, and the morphological variation considerable as well (Figs. 8 and 9, 13). Potentially, many insect taxonomists would agree that such variation in the male (valva shape and vesica diverticulum shape, Fig. 13) and female genitalia (ventral margin of ostium bursae, Figs. 5 and 6) is not intraspecific.

DNA barcodes do not provide a straightforward answer to species delimitation either, ranging between 1.95–4.5% between BINs. Literature on the topic is extensive, and it suffices to say here that if the sequence divergence of lineages exceeds a certain threshold, e.g., 2% after Mutanen et al. (2012) or 3% according to Hebert et al. (2003), then those should be flagged for consideration as distinct species. In the *Aletopus* case, over 3% divergence is present between some lineages. However, in each case a thorough understanding of the taxonomic yardstick is needed, i.e., typical genetic variation in the given lineage. Only after that inferences about the species composition of the genus can reasonably be put forward. Within the Lepidoptera, there are considerable differences in the average minimum distances between families (e.g., Hebert et al., 2003), and in large datasets intraspecific variation has been reported to be as high as 10% (Mutanen et al., 2012). In European Geometridae the mean genetic distance between all species of the family averaged 13.3% (Hausmann, Haszprunar & Hebert, 2011). In a noctuid case the interspecific genetic distances between ingroup taxa within a genus ranged from 1.9–8.2% (Wang et al., 2014).

Finally, the barcode of *Aletopus imperialis* should be assessed to know its genetic profile. This was not possible during the course of this study, due to COVID-19 imposed lockdowns of museums, including the Natural History Museum, London, where the holotype is deposited.

##  Supplemental Information

10.7717/peerj.11613/supp-1 Supplemental Information 1 Raw data: new gene sequences produced in study

10.7717/peerj.11613/supp-2 Supplemental Information 2 ML inference topology for Noctuidae, showing the position of “*Cartaletis*” *dargei* Maximum likelihood inference topology for Noctuidae, based on COI and *wingless* genes, showing the position of “*Cartaletis*” *dargei* within the subfamily Agaristinae. Agaristinae are highlighted with blue. Majority of data are from Zahiri et al. (2013). Numbers above branches are SH-like/UFBoot2 support values.

## Additional Information and Declarations

### Data Availability

The following information was supplied regarding data availability: The new sequences are available at GenBank: MW590952, MW549773, MW548615, MW549774, MW548616, MW549775, MW549776, MW548617, MW590953. The genetic data is also available in the Supplemental File. The COI sequence and other metadata are available at BOLD: DS-AGARIST, “Agaristinae - dargei and imperialis”. doi: dx.doi.org/10.5883/DS-AGARIST.
