# Supplementary material for: Insect taxonomy can be difficult: a noctuid moth (Agaristinae: Aletopus imperialis) and a geometrid moth (Sterrhinae: Cartaletis dargei) combined into a cryptic species complex in eastern Africa (Lepidoptera)
Source: PeerJ. 2021 Jun 25;9:e11613. doi: 10.7717/peerj.11613 (PMC8272464; doi:10.7717/peerj.11613)
Supplement: Supplemental Information 1 [file peerj-09-11613-s001.docx]

**Supplemental File, raw data**

**Manuscript title: Insect taxonomy can be difficult: a noctuid moth (Agaristinae: *Aletopus imperialis*) and a geometrid moth (Sterrhinae: *Cartaletis dargei*) combined into a cryptic species complex in eastern Africa (Lepidoptera)**

Authors: Pasi Sihvonen, Leidys Murillo-Ramos, Niklas Wahlberg, Axel Hausmann, Alberto Zilli, Michael Ochse, Hermann S. Staude

*Genetic data with GenBank accession numbers and sequences in fasta format*

COI-begin MW590952

>BC_ZSM_Lep_58147_dargei_holotype

?AACATTATATTTTATTTTTGGTATTTGATCTGGAATAGTAGGTACTTCTCTTAGATTATTAATTCGGGCAGAATTAGGAACTCCTGGATTATTAATTGGAGATGATCAAATTTATAATACTATTGTAACAGCTCATGCTTTTATTATAATTTTTTTTATAGTTATACCAATCATAATTGGAGGATTTGGTAATTGATTAATTCCCCTAATATTAGGAGCTCCTGATATAGCTTTCCCACGAATAAATAATATAAGCTTTTGACTTCTACCCCCCTCTTTAACCCTTCTTATCTCAAGAAGAATTGTAGAAACTGGAGCAGGTACAGGATGAACAGTATATCCCCCACTTTCATCTAATATTGCTCATAGAGGAAGATCTGTAGATTTAGCTATTTTTTCCCTTCATTTAGCAGGAATCTCTTCAATTTTAGGAGCAATTAATTTTATTACCACAATCATTAATATACGATTAAATAATTTATCTTTTGACCAAATACCCTTATTTGTATGAGCTGTCGGAATTACAGCATTTTTATTATTACTTTCTTTACCAGTATTAGCCGGAGCTATTACTATATTATTAACAGATCGAAATTTAAACACATCTTTTTTTGACCCTGCCGGGGGAGGAGATCCAATTTTATACCAACATTTATTT???????????

COI-begin MW549773

>Sihvonen_DNA_184_Aletopus

??????????????????????????????????????????????????????????attaattcgagcagaattaggaactcctggcctactaattggagatgatcaaatttataatactattgtaacagctcatgcttttattataattttttttatagttataccaatcataattggaggatttggtaattgattaattcccctaatattaggagcccctgatatagctttcccacgaataaataatataagcttttgacttctacccccctccttaaccctccttatctcaagaagaattgtagaaactggagcaggaacaggatgaacagtatatcccccactttcatctaatattgcccacagaggaagatctgtagatttagctattttttcgcttcatttagcaggaatctcttcaattttaggagcaattaattttattaccacaattattaatatacgattgaataatttatcctttgatcaaatacctttatttgtatgagctgtcggaattacagcatttttattattactttctttaccagtattagccggagctattactatattattaacagatcgaaatttaaatacatctttttttgaccctgctggaggaggggatccaattttatatcaacatttatttgattttttgg

Wgl400 MW548615

>Sihvonen_DNA_184_Aletopus

??????????????????????????????????????????????????????????ATTAATTCGAGCAGAATTAGGAACTCCTGGCCTACTAATTGGAGATGATCAAATTTATAATACTATTGTAACAGCTCATGCTTTTATTATAATTTTTTTTATAGTTATACCAATCATAATTGGAGGATTTGGTAATTGATTAATTCCCCTAATATTAGGAGCCCCTGATATAGCTTTCCCACGAATAAATAATATAAGCTTTTGACTTCTACCCCCCTCCTTAACCCTCCTTATCTCAAGAAGAATTGTAGAAACTGGAGCAGGAACAGGATGAACAGTATATCCCCCACTTTCATCTAATATTGCCCACAGAGGAAGATCTGTAGATTTAGCTATTTTTTCGCTTCATTTAGCAGGAATCTCTTCAATTTTAGGAGCAATTAATTTTATTACCACAATTATTAATATACGATTGAATAATTTATCCTTTGATCAAATACCTTTATTTGTATGAGCTGTCGGAATTACAGCATTTTTATTATTACTTTCTTTACCAGTATTAGCCGGAGCTATTACTATATTATTAACAGATCGAAATTTAAATACATCTTTTTTTGACCCTGCTGGAGGAGGGGATCCAATTTTATATCAACATTTATTTTGATTTTTTGG

COI-begin HM376557

>BC_ZSM_Lep_20051_Aletopus

?AACATTATATTTTATTTTTGGTATTTGATCTGGAATAGTAGGTACTTCTCTTAGATTATTAATTCGAGCAGAATTAGGAACTCCTGGCCTGCTAATTGGAGATGATCAAATTTATAATACTATTGTAACAGCTCATGCTTTTATTATAATTTTTTTTATAGTTATGCCAATCATAATTGGAGGATTTGGTAATTGATTAATTCCCCTAATATTAGGAGCCCCTGATATAGCTTTTCCACGAATAAATAATATAAGCTTTTGACTTCTACCCCCCTCCTTAACCCTCCTTATCTCAAGAAGAATTGTAGAAACTGGAGCAGGTACAGGATGAACAGTATATCCCCCACTTTCATCTAATATTGCCCACAGAGGAAGATCTGTAGATTTAGCTATTTTTTCACTTCATTTAGCAGGAATCTCTTCAATTTTAGGAGCAATTAATTTTATTACCACAATTATTAATATACGATTGAATAATTTATCTTTTGATCAAATACCTTTATTTGTATGAGCTGTCGGAATTACAGCATTTTTATTATTACTTTCTTTACCAGTATTAGCCGGAGCTATTACTATATTATTAACAGATCGGAATTTAAATACATCTTTTTTTGACCCTGCTGGAGGAGGGGATCCAATTTTATACCAACATTTATTT???????????

COI-begin MW549774

>Sihvonen_DNA_185_Aletopus

?????????????????????????????????????????GGTACTTCTCTTAGATTGTTAATTCGAGCAGAATTAGGAACTCCTGGACTACTAATTGGAGATGATCAAATTTATAATACTATTGTAACAGCTCATGCTTTTATTATAATTTTTTTTATGGTTATACCAATCATAATTGGAGGATTTGGTAATTGATTAATTCCCTTAATATTAGGAGCCCCTGACATAGCTTTCCCACGAATAAATAACATAAGCTTTTGACTTTTACCCCCCTCTTTAACCCTTCTTATCTCAAGAAGAATTGTAGAAACTGGAGCAGGTACAGGATGAACAGTATACCCCCCACTTTCATCTAATATTGCCCATAGAGGAAGATCTGTAGATTTAGCTATTTTCTCGCTTCATTTAGCAGGAATCTCTTCAATTTTAGGAGCAATTAATTTTATTACTACAATTATTAATATACGATTGAATAATTTATCTTTTGATCAAATACCTTTATTTGTATGATCTGTCGGAATTACAGCATTTTTATTATTACTTTCTTTACCAGTATTAGCCGGAGCTATTACTATATTATTAACAGATCGAAATTTAAATACATCTTTTTTTGACCCTGCTGGAGGGGGGGATCCAATTTTATATCAACATTTGTTTTGATTTTTTGG

>ALETO003-20_Sihvonen_188_Cartaletis_dargei

Wgl400 MW548616

>Sihvonen_DNA_185_Aletopus

GGACCGCTTCGACGGCGCGTCGCGGGTCATGATGCCCAACACTGACCTGGAGGCGCCGGTACAGCGGAACGACGCGGCACCGCACAGAGTGCCGCGAAGAGACCGGTATCGATTCCAACTGCGACCTCACAATCCAGACCACAAATCGCCGGGGGCGAAAGACTTAGTTTACTTAGAGTCATCTCCGGGCTTCTGTGAAAAGAACCCGAGGCTAGGCATCCCCGGCACACACGGGCGCGCCTGCAATGACACGAGCATCGGGGTGGACGGCTGCGACCTCATGTGCTGCGGCCGGGGCTACCGGACGGAGACCATGTTCGTGGTGGAGCGGTGCAAC

COI-begin MW549775

>Sihvonen_DNA_188_Aletopus

?????????????????????????????????????????????CTTCTCTTAGATTGTTAATTCGAGCAGAATTAGGAACTCCTGGACTACTAATTGGAGATGATCAAATTTATAATACTATTGTAACAGCTCATGCTTTTATTATAATTTTTTTTATGGTTATACCAATCATAATTGGAGGATTTGGTAATTGATTAATTCCCTTAATATTAGGAGCCCCTGACATAGCTTTCCCACGAATAAATAACATAAGCTTTTGACTTTTACCCCCCTCTTTAACCCTTCTTATCTCAAGAAGAATTGTAGAAACTGGAGCAGGTACAGGATGAACAGTATACCCCCCACTTTCATCTAATATTGCCCATAGAGGAAGATCTGTAGATTTAGCTATTTTCTCGCTTCATTTAGCAGGAATCTCTTCAATTTTAGGAGCAATTAATTTTATTACTACAATTATTAATATACGATTGAATAATTTATCTTTTGATCAAATACCTTTATTTGTATGATCTGTCGGAATTACAGCATTTTTATTATTACTTTCTTTACCAGTATTAGCCGGAGCTATTACTATATTATTAACAGATCGAAATTTAAATACATCTTTTTTTGACCCTGCTGGAGGGGGGGATCCAATTTTATATCAACATTTGTTTTGATTTTTTGG

COI-begin MW549776

>Sihvonen_DNA_190_Aletopus

GAACATTATATTTTATTTTTGGTATTTGATCTGGAATAGTAGGTACTTCTCTTAGATTGTTAATTCGAGCAGAATTAGGAACTCCTGGACTACTAATTGGAGATGAACAAATTTATAATACTATTGTAACAGCTCATGCTTTTATTATAATTTTTTTTATGGTTATACCAATCATAATTGGAGGATTTGGTAATTGATTAATTCCCTTAATATTAGGAGCCCCTGACATAGCTTTCCCACGAAAAAATAACATAAGCTTTTGACTTTTACCCCCCTCTTTAACCCTCCTTATCTCAAGAAGAATTGTAAAAACTGGAGCAGGGACAGGATGAACAGTATATCCCCCACTTTCATCTAATATTGCCCATAGAGGAAGATCTGTAGATTTAGCTATTTTCTCGCTTCATTTAGCAGGAATCTCTTCAATTTTAGGAGCAATTAATTTTATTACTACAATTATTAATATACGATTGAATAATTTATCTTTTGATCAAATACCTTTATTTGTATGAGCTGTCGGAATTACAGCATTTTTATTATTACTTTCTTTACCAGTATTGGCCGGAGCTATTACTATATTATTAACAGATCGAAATTTAAATACATCTTTTTTTGATCCTGCTGGAGGAGGAGATCCAATTTTATATCAACATTTATTTTGATTTTTTGG

Wgl400 MW548617

>Sihvonen_DNA_190_Aletopus

?????????????????CGTCGCGGGTCATGATGCCCAACACTGACCTGGAGGCGCCGGCACAGCGGAACGACGCGGCACCGCACAGAGTGCCGCGAAGAGACCGGTATCGATTCCAACTGCGACCTCACAATCCAGACCACAAATCGCCGGGGGCGAAAGACTTAGTTTACTTAGAGTCATCTCCGGGCTTCTGTGAAAAGAACCCGAGGCTAGGCATCCGCGGCACACACGGGCGCGCCTGCACTGACACGAGCATCGGGGTGGACGGCTGCGACCTCATGTGCTGCGGCCGGGGCTACCGGACGGAGACCATGTTCGTGGTGGAGCGGCGCAAC

COI-begin MW590953

>BC_ZSM_Lep_42575_Aletopus

?AACATTATATTTTATTTTTGGTATTTGATCTGGAATAGTAGGTACTTCTCTTAGATTGTTAATTCGAGCAGAATTAGGAACTCCTGGACTACTAATTGGAGATGATCAAATTTATAATACTATTGTAACAGCTCATGCTTTTATTATAATTTTTTTTATGGTTATACCAATCATAATTGGAGGATTTGGTAATTGATTAATTCCCTTAATATTAGGAGCCCCTGACATAGCTTTCCCACGAATAAATAACATAAGCTTTTGACTTTTACCCCCCTCTTTAACCCTTCTTATCTCAAGAAGAATTGTAGAAACTGGAGCAGGTACAGGATGAACAGTATACCCCCCACTTTCATCTAATATTGCCCATAGAGGAAGATCTGTAGATTTAGCTATTTTCTCGCTTCATTTAGCAGGAATCTCTTCAATTTTAGGAGCAATTAATTTTATTACTACAATTATTAATATACGATTGAATAATTTATCTTTTGATCAAATACCTTTATTTGTATGATCTGTCGGAATTACAGCATTTTTATTATTACTTTCTTTACCAGTATTAGCCGGAGCTATTACTATATTATTAACAGATCGAAATTTAAATACATCTTTTTTTGACCCTGCTGGAGGGGGGGATCCAATTTTATATCAACATTTGTTT???????????
