# Supplementary figures and images for: Insect taxonomy can be difficult: a noctuid moth (Agaristinae: Aletopus imperialis) and a geometrid moth (Sterrhinae: Cartaletis dargei) combined into a cryptic species complex in eastern Africa (Lepidoptera)
Source: PeerJ. 2021 Jun 25;9:e11613. doi: 10.7717/peerj.11613 (PMC8272464; doi:10.7717/peerj.11613)

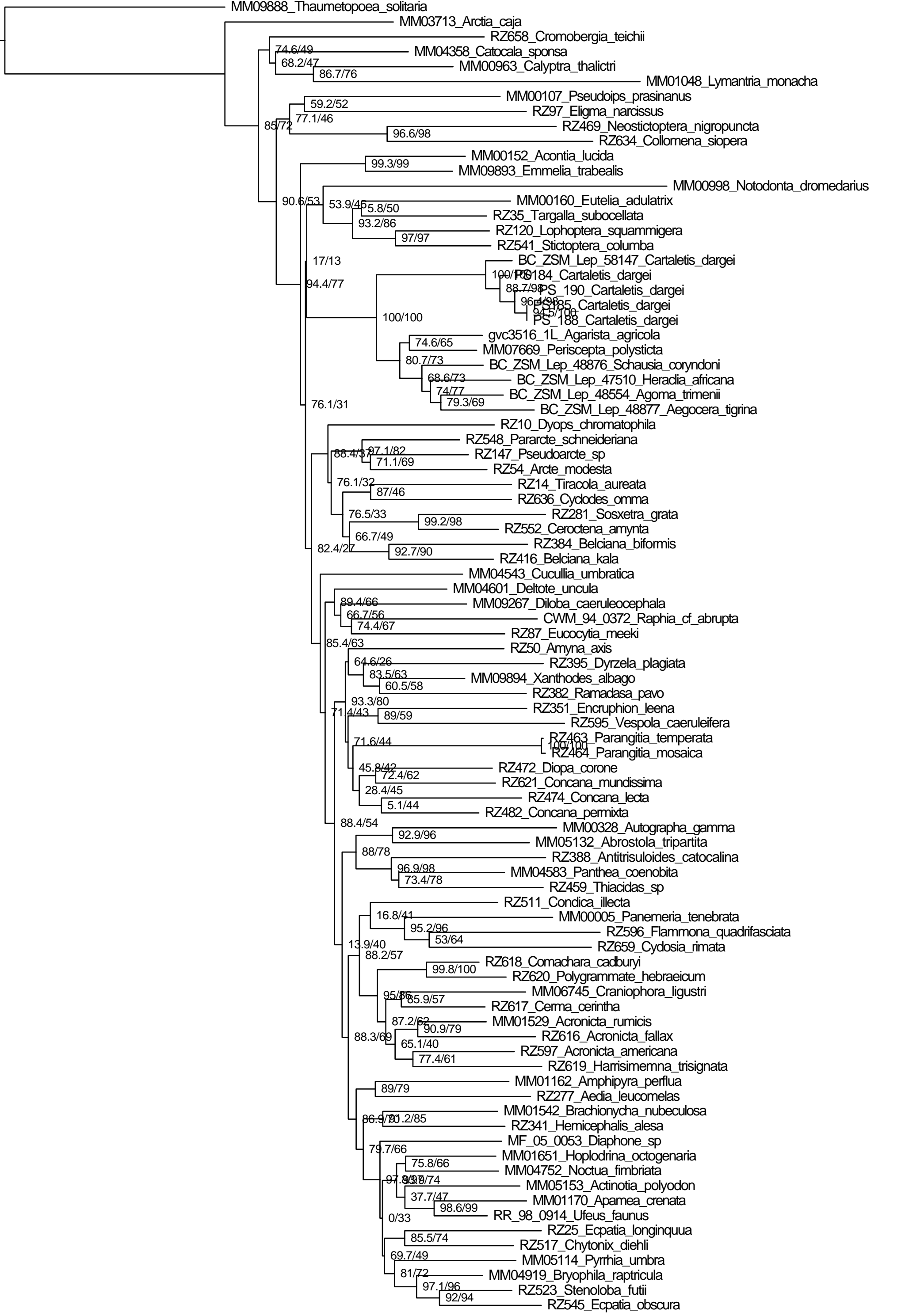

0.06

Supplement: Supplemental Information 2 — Maximum likelihood inference topology for Noctuidae, based on COI and wingless genes, showing the position of “Cartaletis” dargei within the subfamily Agaristinae. Agaristinae are highlighted with blue. Majority of data are from Zahiri et al. (2013). Numbers above branches are SH-like/UFBoot2 support values. [file peerj-09-11613-s002.pdf]
